# Genome-wide analysis of Hsp40 and Hsp70 gene family in four cotton species provides insights into their involvement in response to *Verticillium dahliae* and abiotic stress

**DOI:** 10.3389/fgene.2023.1120861

**Published:** 2023-01-26

**Authors:** Xin Zhou, Ling Su, Rui Tang, Yuxuan Dong, Fei Wang, Rong Li, Quanliang Xie, Xianliang Zhang, Guanghui Xiao, Hongbin Li

**Affiliations:** ^1^ Key Laboratory of Xinjiang Phytomedicine Resource and Utilization of Ministry of Education, Key Laboratory of Oasis Town and Mountain-basin System Ecology of Xinjiang Production and Construction Corps, College of Life Sciences, Shihezi University, Shihezi, China; ^2^ College of Life Sciences, Shaanxi Normal University, Xi’an, China; ^3^ National Key Laboratory of Cotton Biology, Institute of Cotton Research (CAAS), Anyang, China

**Keywords:** cotton, HSP40 gene family, HSP70 gene family, *Verticillium* wilt resistance, abiotic stress response

## Abstract

**Introduction:** Cotton is an important economic crop to provide natural fibers as raw materials to textile industry, and is significantly affected by biotic and abiotic stress during the whole growth stage, in which *Verticillium* wilt (VW) caused by *Verticillium dahliae* is one of the most destructive disease to lead to a significant yield reduction. Heat shock proteins (Hsps) are important molecular chaperones, and play crucial roles in plant growth, development, resistance to biotic and abiotic stress. Hsp40 and Hsp70 are two key Hsps in cell chaperone network, however, the function and regulatory mechanism of Hsp40 and Hsp70 members in VW resistance and abiotic stress in cotton are largely unknown.

**Methods and Results:** Herein, a systematic and comprehensive analysis of Hsp40s and Hsp70s in four cotton species of *Gossypium arboretum*,* G. raimondii*, *G. hirsutum*, and *G. barbadense* were performed. A total of 291 Hsp40s and 171 Hsp70s identified in four Gossypium species. Sequence analysis revealed that all Hsp40 proteins contained J domain that provides the binding sites to Hsp70. Protein-protein interaction prediction analysis displayed that GhHsp40-55 might interact with GhHsp70-2 and GhHsp70-13, suggesting their potential function as protein complex. Promoter cis-acting element analysis demonstrated that multiple cis-elements related to disease and stress response consists in GhHsp40 and GhHsp70 promoters. Further expression analysis showed that eight GhHsp40s (Hsp40-2,4,8,11,20,23,53,55) and seven GhHsp70s (Hsp70-2,3,6,8,13,19,22) were up-regulated after *V. dahliae* infection. In addition, five GhHsp40s (Hsp40-2,8,11,53,55) and four GhHsp70s (Hsp70-3,6,8,13) were up-regulated after salt treatment, six GhHsp40s (Hsp40-4,11,20,23) and three GhHsp70s (Hsp70-2,8,19) were up-regulated after drought treatment, four GhHsp40s (Hsp40-2,11,20,23) and four GhHsp70s (Hsp70-3,6,19,22) were up-regulated after temperature treatment, suggesting these Hsps have possible important function in the process of abiotic stress response.

**Discussion:** Our results lay a foundation for understanding the function of Hsp40 and Hsp70 in the resistance against *V. dahliae* and abiotic stress, and elucidating the regulatory mechanism of the protein complex, evolution and molecular mechanism under stress.

## Introduction

Since plants do not have the ability to escape adverse environment, their growth, development and production are seriously affected by abiotic and biological stresses (Guo et al., 2021). In recent decades, biological and abiotic threats have reduced crop yield potential by about 70% (Katiyar-Agarwal et al., 2006; Nagaraju et al., 2020), causing serious economic losses to global crop production (Panzade et al., 2021).

Cotton (*Gossypium spp.*) is an important economic crop in the world, providing the most important natural fiber for the textile industry (Zhao et al., 2021). Studies show that *Verticillium* wilt (VW) is a typical fungal disease caused by *V. dahliae* Kleb, which seriously affects the yield and quality of cotton (Zhang et al., 2014; Zhao et al., 2021). VW pathogens usually exist in the soil in the form of microsclerotia, invading xylem and vascular tissue from the root of the host plant, and then spreading to the aboveground part, resulting in the leaves to lose green, necrosis or wilting, leaf and boll shedding until plant death (Zhou et al., 2017). In addition, with global climate change and environmental damage, cotton growth and development are also affected by various environmental pressures, such as drought, salt, alkali, low and high temperature abiotic stress (Parida et al., 2007). These biotic and abiotic stresses affect the growth and development process of cotton by affecting its physiological and metabolic reactions, causing irreversible damage or even death, and ultimately resulting in cotton yield reduction.

In order to ensure successful reproduction, plants have evolved a variety of defense mechanisms against various biotic and abiotic stresses, such as the ability to detect pathogens and induce rapid immune response through innate immune surveillance systems (Nejat & Mantri, 2017; Saijo et al., 2018). In addition, transcription factor (TF) is described as a group of proteins that participate in the regulation of specific gene expression by attaching to the promoter or enhance region of DNA. At the same time, they produce a group of proteins called stress proteins, which need to have a unique three-dimensional structure in order to function. Chaperones, also known as heat shock proteins (Hsps), protect the three-dimensional structure of a large number of proteins under different stress conditions. They are a very large family of proteins that are present in all living organisms. As a template, chaperone protein can fold correctly and form a functional three-dimensional structure immediately during and after synthesis, so that enough chaperone cells can be synthesized to better protect proteins and other structures and survive under stress conditions (Liberek et al., 2008). According to its molecular weight and function, *Hsps* can be divided into several families, including *Hsp100*, *Hsp90*, *Hsp70* (also named DnaK in *Escherichia coli*), *Hsp60*, *Hsp40* (also referred to as DnaJ) and low molecular weight *Hsps* (Guo et al., 2021). Heat shot protein 70s (Hsp70s) is a key component of the cell chaperone network (Qiu et al., 2006). The expression of Hsp70 family is either induced by various stresses or constitutive. For example, *Hsp70-2* and *Hsc70t* have been shown to play a special role in sperm development (Dix et al., 1997; Eddy, 1999). In *Arabidopsis thaliana*, the mutant *cpHsc70-1* showed a defective phenotype when the germinated seeds were subjected to heat stress (Su and Li, 2008). Heat stress induced the expression of *MsHsp70*, and transgenic lines of arabidopsis overexpressing *MsHsp70* enhanced their tolerance to drought stress (Li et al., 2017). Under stress conditions, *Hsp70* also plays an important role in wheat (Duan et al., 2011), cucumber (Li et al., 2014), pepper (Guo et al., 2014), rubber trees and other plants (Zhang et al., 2009). Hsp40, also known as DnaJ protein or J protein (Georgopoulos et al., 1980), is usually composed of a J-domain. As a co-partner of Hsp70, Hsp40 can strictly regulate ATP hydrolysis. Previous studies have shown that *Hsp70* and *Hsp40* are also involved in disease resistance of various plants, such as MP interacting protein 1 *NtMPIP1* (Shimizu et al., 2009), *GmHSP40.1* (Liu and Whitham, 2013), and rice DnaJ gene *OsDjA6* (Zhong et al., 2018).

Hsps are evolutionarily conservative in many plants. In recent years, several studies have been conducted at the genome level to identify the function of *Hsps* genes in many plants (Krishna and Gloor, 2001; Zhang et al., 2014), therefore, the identification of *Hsps* genes are particularly important for further molecular function studies. With the development of sequencing technology, the genome sequencing of many plants has been completed, which greatly promotes the identification and phylogenetic analysis of plant homologous genes (Wang et al., 2016). So far, studies have reported 89 *Hsp40s* and 18 *Hsp70s* from Arabidopsis; 104 *Hsp40s* and 32 *Hsp70s* were identified in rice; 145 *Hsp40s* and 34 *Hsp70s* were identified in poplar; 91 *Hsp40s* and 27 *Hsp70s* were identified in eucalyptus (Zhang et al., 2014; Yer et al., 2018). However, the studies on the identification of Hsp40s and Hsp70s in cotton have not been reported yet. Based on the whole genome sequences of *Gossypium arboretum*, *G. raimondii*, *G. hirsutum*, and *G. barbadense*, we used bioinformatics methods to identify members of the *Hsp40s* and *Hsp70s* gene families in four cotton varieties. The number, chromosome location, phylogenetic evolution, expression pattern and interaction of *Hsp40s* and *Hsp70s* gene family members in cotton genome were analyzed at the genome level, providing reference for further research on the biological functions of *Hsp40s* and *Hsp70s* genes in cotton stress and verticillium wilt resistance.

## Materials and methods

### Genome *databases*

The genome sequence of *Arabidopsis thaliana* used in this study was downloaded from The Arabidopsis Information Resource (TAIR) database (Lamesch et al., 2012). The genome sequences of *G. hirsutum* genome (NDM8), *G. raimondii* (JGI_v2.1), *G. arboreum* (CRI_v3.0), *G. barbadense* (HAU v1) were downloaded from Cotton FGD (https://cottonfgd.org/) and CottonGEN (https://cottongen.org/).

### Identification *of Hsp40* and *Hsp70* genes in cotton genome

In this research, we used “Heat Shock Protein Database Information Resource” (HSPIR) (https://pdslab.biochem.iisc.ernet.in/hspir/index.php) in order to identify the potential Hsp40s/Hsp70s in the *G. hirsutum*., *G. raimondii*, *G. arboreum* and *G. barbadense* genomes. Only the genes that were homologous with Hsp40s/Hsp70s (Evalue ≤ 1.0 × 10^–5^) and contain the Dna J domains were considered as *GhHsp40s/Hsp70s*, *GaHsp40s/Hsp7*0s, *GrHsp40s/Hsp70s* and *GbHsp40s/Hsp70s*. We used the general feature format (GFF) file of the genomes to determine the relative position of *Hsp40s/Hsp70s* on chromosomes, and visualized the locations with the online software MG2C (Jiangtao et al., 2015). Furthermore, the gene structures of *Hsp40s/Hsp70s* were also analyzed according to the GFF files, and the “exon-intron” structure was shown by the Gene Structure Display Server (GSDS 2.08).

### Sequence *analysis* of *Hsp40s/Hsp70s*

Protein motif analysis was performed using MEME9 with a maximum of eight motifs and using other default parameters. The physicochemical properties, including molecular weight (MW), isoelectric point (pI), instability index, and grand average of hydropathicity (GRAVY), were analyzed using the online software ExPASy ProtParam tool (https://web.expasy.org/cgi-bin/protparam/protparam) in *GhHsp40s/Hsp70s*, *GaHsp40s/Hsp70s*, *GrHsp40s/Hsp70s*, and *GbHsp40s/Hsp70s*, respectively.

### Phylogenetic tree construction of *Hsp40s/Hsp70s*

The Hsp40s/Hsp70s protein sequences of *G. hirsutum*., *G. raimondii*, *G. arboreum*, *G. barbadense* and *A. thaliana* were aligned using ClustalW (https://www.genome.jp/tools-bin/clustalw), and the phylogenetic tree was constructed using the neighbor-joining algorithm with MEAG 7.0 (Kumar et al., 2016). Confidence values were obtained with bootstrapping with 1,000 replications.

### Spatial and temporal expression *analysis* of *GhHSP40s/Hsp70s* genes

To illustrate the spatial and temporal expression patterns of *GhHsp40s/Hsp70s*, the RNA-seq data of abiotic stress, and *Verticillium* wilt expression data were downloaded from NCBI (accession NO. PRJNA248163, PRJNA490626, PRJNA680449) (Zhang et al., 2014; Hu et al., 2019). The expression levels of *GhHsp40s/Hsp70s* genes were visualized using the R language package Pheatmap (https://cran.r-project.org/web/packages/pheatmap/).

### Statistical *analysis*

Mean values and standard errors were calculated with Microsoft Excel software. Student’s t*-*test was completed with the SPSS 23.0 program to assess the significance of any differences between the control and treated samples or between time points. The threshold for significance was set at *p* < .01.

## Results

### Genome-wide *identifification* of *Hsp40* and *Hsp70* gene family members in cotton species

To identify members of the *Hsp40* and *Hsp70* gene families in cotton, this study used The Arabidopsis Information Resource (TAIR, https://www.arabidopsis.org/) database (Lamesch et al., 2012) to downloaded AtHsp40s and AtHsp70s protein sequences which are used to retrieve the protein Sequences of *G. hirsutum* (NDM8), *G. raimondii* (JGI_v2.1), *G. arboreum* (cri_V0.3), *G. hirsutum* (NDM8), *G. raimondii* (JGI_v2.1), *G. Arboreum* (cri_V0.3). HMMER 3.0 software (on a Windows system) was utilized to search these cotton databases using DnaJ and DnaK domain as query domain. Finally, we identified 291 *Hsp40s* and 171 *Hsp70s*, including 93 *GhHsp40s*, 91 *GbHsp40s*, 45 *GaHsp40s*, and 62 *GrHsp40s*, as well as 77 *GhHsp70s*, 44 *GbHsp70s*, 25 *GaHsp70s*, and 25 *GrHsp70s* (Supplementary Table S1).

### Physio-*biochemical* properties analysis

Then we analyzed the physical and chemical properties of the members of the *Hsp40* and *Hsp70* genes families in upland cotton, and found that the amino acid number of the protein encoded by the members of the two families has a large span. The longest *Hsp40* was 1,404 amino acids (*GhHsp40-42* and *GhHsp40-59*) and the shortest was 143.

Amino acids (*GhHsp40-21*). The longest *Hsp70* was 886 amino acids (*GhHsp70-11*) and the shortest was 143 amino acids (*GhHsp70-21*). The molecular weight of *GhHsp40s* ranges from 15.61 kDa (*GhHsp40-21*) to 154.43 kDa (*GhHsp40-42* and *GhHsp40-59*). The molecular weight of *GhHsp70s* ranges from 6.79 kDa (*GhHsp70-21*) to 98.69 kDa (*GhHsp70-56*). Based on isoelectric point (PI) analysis, *GhHsp70s* were acidic proteins with PI less than 7.0 (average 5.38). In contrast, *GhHsp40s* were predicted to encode proteins in excess of 7.0 (average 7.71) and to be alkaline*.* Hydrophilic analysis (GRAVY) found that all *GhHsp40s* and *GhHsp70s* were less than 0, indicating that GhHsp40s and GhHsp70s are hydrophilic proteins. According to the analysis of instability index, the mean instability index of *GhHsp40s* protein is 41.47, among which 53 are less than 40.0 and 41 are greater than 40.0. The instability index values of three *GhHsp40s* were greater than 60.0 (*GhHsp40-4*, *GhHsp40-38*, *GhHsp40-61* and *GhHsp40-80*). The mean instability index of GhHsp70s protein was 36.62, of which 55 GhHsp70s were less than 40.0 and 23 GhHsp70s were greater than 40.0. Detailed physical and chemical properties are shown in Tables 1, 2.

**TABLE 1:** Information of *GhHsp40* gene family in cotton.

Gene name	Accession number	Number of amino acid	Molecular weight	Theoretical pI	Instability index	Aliphatic index	Grand average of hydropathicity	Chr	Position
GhHsp40-1	Ghir_A06G018560.1	418	46505.6	6.12	31.43	64.14	−0.747	Ghir_A06	118862803–118865613
GhHsp40-2	Ghir_D01G023660.1	340	37318.55	9.23	35.33	65.97	−0.634	Ghir_D01	62920176–62924378
GhHsp40-3	Ghir_D11G008300.1	423	47399.48	7.23	38.74	59.91	−0.85	Ghir_D11	6740855–6744528
GhHsp40-4	Ghir_D02G012440.1	148	17127.43	5.92	61	76.96	−0.803	Ghir_D02	39900716–39901550
GhHsp40-5	Ghir_D02G012440.6								
GhHsp40-6	Ghir_D02G012440.3								
GhHsp40-7	Ghir_D02G012440.4								
GhHsp40-8	Ghir_A08G012700.1	342	38899.2	6.4	40.7	74.94	−0.628	Ghir_A08	90517348–90521329
GhHsp40-9	Ghir_D02G012440.2								
GhHsp40-10	Ghir_A06G003370.6	411	45699.97	7.88	40.51	71.92	−0.297	Ghir_A06	4263575–4270663
GhHsp40-11	Ghir_A06G011640.1	507	54190.95	8.91	35.92	68.11	−0.408	Ghir_A06	55723023–55731818
GhHsp40-12	Ghir_D07G012170.1	416	46199.26	5.87	34.19	66.35	−0.684	Ghir_D07	16765193–16767740
GhHsp40-13	Ghir_D02G017540.1	287	32080.77	9.3	41.59	84.6	−0.571	Ghir_D02	60601174–60603919
GhHsp40-14	Ghir_A06G003370.1	447	49681.03	7.91	39.78	67.02	−0.493	Ghir_A06	4263575–4270663
GhHsp40-15	Ghir_D08G013380.2	271	30719.13	5.23	39.35	80.96	−0.362	Ghir_D08	46247844–46251903
GhHsp40-16	Ghir_A06G003370.2	456	50758.23	7.91	39.47	67.41	−0.491	Ghir_A06	4263575–4270663
GhHsp40-17	Ghir_D08G013380.1	343	38977.41	6.35	39.31	76.15	−0.595	Ghir_D08	46247844–46251903
GhHsp40-18	Ghir_A06G003370.3	480	53628.94	8.15	38.95	73.79	−0.392	Ghir_A06	4263575–4270663
GhHsp40-19	Ghir_D12G007580.1	177	19011.09	9.48	51.59	87.97	−0.237	Ghir_D12	15748808–15749341
GhHsp40-20	Ghir_A05G001150.1	337	37265.17	8.97	36.28	69.41	−0.71	Ghir_A05	1256284–1259560
GhHsp40-21	Ghir_A12G008150.1	143	15611.23	9.48	45.31	107.48	−0.162	Ghir_A12	32460209–32460640
GhHsp40-22	Ghir_A06G003370.5	480	53628.94	8.15	38.95	73.79	−0.392	Ghir_A06	4263575–4270663
GhHsp40-23	Ghir_A03G007540.1	354	39930.05	9.15	42.89	73.59	−0.774	Ghir_A03	17010055–17014081
GhHsp40-24	Ghir_A06G003370.7	411	45699.97	7.88	40.51	71.92	−0.297	Ghir_A06	4263575–4270663
GhHsp40-25	Ghir_A11G011860.1	344	38771.07	6.11	36.39	79.33	−0.524	Ghir_A11	11843649–11860959
GhHsp40-26	Ghir_A11G011860.2	344	38771.07	6.11	36.39	79.33	−0.524	Ghir_A11	11855835–11860959
GhHsp40-27	Ghir_A11G011860.3	245	27534.08	5.12	43.72	77.18	−0.539	Ghir_A11	11856525–11860959
GhHsp40-28	Ghir_A13G022970.1	1287	141203.34	6.32	48.51	65.54	−0.619	Ghir_A13	106789915–106797363
GhHsp40-29	Ghir_D03G010050.1	354	39773.91	9.15	43.26	74.12	−0.753	Ghir_D03	35405754–35409583
GhHsp40-30	Ghir_A04G009850.1	343	37808.18	9.1	39.98	62.83	−0.652	Ghir_A04	73367279–73370751
GhHsp40-31	Ghir_A04G009850.2	266	28800.02	9.11	44.08	64.14	−0.462	Ghir_A04	73367279–73370751
GhHsp40-32	Ghir_A04G009850.3	343	37808.18	9.1	39.98	62.83	−0.652	Ghir_A04	73367279–73370751
GhHsp40-33	Ghir_A04G009850.4	266	28800.02	9.11	44.08	64.14	−0.462	Ghir_A04	73367279–73370751
GhHsp40-34	Ghir_A02G015750.1	357	38511.57	9.18	43.9	69.61	−0.346	Ghir_A02	103092402–103097140
GhHsp40-35	Ghir_D06G004990.1	336	36855.25	8.62	38.25	64.73	−0.765	Ghir_D06	6999139–7002587
GhHsp40-36	Ghir_A05G016400.1	344	38004.26	9.13	38.1	67.76	−0.653	Ghir_A05	15517231–15519777
GhHsp40-37	Ghir_D06G019440.1	418	46530.59	6.06	35.02	62.75	−0.756	Ghir_D06	61633441–61636250
GhHsp40-38	Ghir_D06G004830.1	354	39130.31	9.18	62.42	67.71	−0.687	Ghir_D06	6736388–6737681
GhHsp40-39	Ghir_D01G001970.1	348	38639.24	8.66	32.73	81.24	−0.395	Ghir_D01	1576299–1578283
GhHsp40-40	Ghir_D09G012410.1	1354	148145.79	5.64	43.81	61.29	−0.648	Ghir_D09	39923973–39935520
GhHsp40-41	Ghir_D06G019440.2	337	37335.35	5.97	37.27	64.48	−0.634	Ghir_D06	61633492–61636250
GhHsp40-42	Ghir_A09G012940.1	1404	154429.32	5.98	44.81	59.94	−0.718	Ghir_A09	67361938–67374345
GhHsp40-43	Ghir_D13G023610.1	1287	141496.56	6.23	48.96	65.16	−0.629	Ghir_D13	61510482–61518051
GhHsp40-44	Ghir_A05G029190.1	337	37215.21	5.85	39.17	65.93	−0.605	Ghir_A05	34107181–34113682
GhHsp40-45	Ghir_A12G001520.1	281	30227.56	8.64	37.04	78.33	−0.306	Ghir_A12	2015359–2016312
GhHsp40-46	Ghir_A05G001150.1	337	37265.17	8.97	36.28	69.41	−0.71	Ghir_A05	1256284–1259560
GhHsp40-47	Ghir_A05G001150.2	337	37265.17	8.97	36.28	69.41	−0.71	Ghir_A05	1256284–1259560
GhHsp40-48	Ghir_D02G012440.7								
GhHsp40-49	Ghir_A11G008330.1	411	46921.75	7.58	40.15	68.98	−0.677	Ghir_A11	7358226–7361961
GhHsp40-50	Ghir_A11G008330.2	422	47213.27	6.97	37.27	61	−0.829	Ghir_A11	7358226–7361961
GhHsp40-51	Ghir_D05G001290.1	337	37269.15	8.63	35.41	68.84	−0.714	Ghir_D05	1191063–1193609
GhHsp40-52	Ghir_D08G005970.1	444	48419.94	9.04	36.84	75.05	−0.421	Ghir_D08	6894751–6901478
GhHsp40-53	Ghir_A03G016270.1	287	32099.78	9.36	42.71	83.24	−0.59	Ghir_A03	101288904–101291666
GhHsp40-54	Ghir_D12G001560.1	435	47429.13	9.31	36.66	79.31	−0.388	Ghir_D12	1877205–1880794
GhHsp40-55	Ghir_D04G014090.1	343	37820.22	9.19	44.73	65.98	−0.633	Ghir_D04	46587713–46591189
GhHsp40-56	Ghir_A07G012090.1	413	46057.09	5.87	34.01	67.05	−0.705	Ghir_A07	21505958–21508165
GhHsp40-57	Ghir_A05G007300.1	1217	133545.64	8.18	49.53	65.09	−0.686	Ghir_A05	6676721–6683582
GhHsp40-58	Ghir_D05G024010.1	280	30945.48	9.57	47.04	76.89	−0.448	Ghir_D05	21859559–21863661
GhHsp40-59	Ghir_A09G012940.1	1404	154429.32	5.98	44.81	59.94	−0.718	Ghir_A09	67361938–67374345
GhHsp40-60	Ghir_D08G005970.1	444	48419.94	9.04	36.84	75.05	−0.421	Ghir_D08	6894751–6901478
GhHsp40-61	Ghir_D02G012440.1	148	17127.43	5.92	61	76.96	−0.803	Ghir_D02	39900716–39901550
GhHsp40-62	Ghir_A08G012700.2	243	27577.21	5.32	47.17	70.62	−0.612	Ghir_A08	90517363–90521343
GhHsp40-63	Ghir_A01G002030.1	348	38561.07	8.99	34.8	78.99	−0.41	Ghir_A01	1844253–1846622
GhHsp40-64	Ghir_A09G010890.1	417	46275.33	5.78	39.64	64.53	−0.731	Ghir_A09	63753113–63756731
GhHsp40-65	Ghir_A06G004780.1	257	28136.01	6.85	56.12	75.49	−0.536	Ghir_A06	7884198–7885883
GhHsp40-66	Ghir_D03G003790.1	443	47908.6	9.39	43.9	74.79	−0.355	Ghir_D03	4462430–4467251
GhHsp40-67	Ghir_A06G004950.1	267	29467.77	7.69	43.33	56.63	−0.882	Ghir_A06	8114097–8117483
GhHsp40-68	Ghir_D05G007350.1	1217	133378.29	7.34	49.87	65.41	−0.676	Ghir_D05	5954069–5961562
GhHsp40-69	Ghir_A01G000580.1	401	45420.12	9.33	59.39	63.14	−1.004	Ghir_A01	454482–456926
GhHsp40-70	Ghir_A01G022120.1	340	37322.55	9.23	35.33	64.53	−0.648	Ghir_A01	117424681–117427892
GhHsp40-71	Ghir_D06G003390.5	456	50631.9	6.8	40.93	65.9	−0.516	Ghir_D06	4035491–4042307
GhHsp40-72	Ghir_A03G010330.1	525	57954.97	9.31	36.67	77.62	−0.313	Ghir_A03	46286381–46298115
GhHsp40-73	Ghir_D06G003390.6	447	49554.69	6.8	41.26	65.48	−0.519	Ghir_D06	4035491–4042307
GhHsp40-74	Ghir_D04G014090.2	266	28800.05	9.24	50.52	67.82	−0.446	Ghir_D04	46587713–46591189
GhHsp40-75	Ghir_D06G003390.3	480	53502.6	7.14	40.33	72.35	−0.416	Ghir_D06	4035348–4042307
GhHsp40-76	Ghir_D11G011810.2	344	38860.21	6.05	37.99	80.17	−0.529	Ghir_D11	10611312–10616648
GhHsp40-77	Ghir_A05G024150.1	280	31082.64	9.53	47.36	74.11	−0.475	Ghir_A05	24090835–24092470
GhHsp40-78	Ghir_D06G003390.4	480	53502.6	7.14	40.33	72.35	−0.416	Ghir_D06	4035491–4042307
GhHsp40-79	Ghir_D11G011810.1	344	38772.14	6.19	37.99	80.47	−0.513	Ghir_D11	10611394–10634402
GhHsp40-80	Ghir_D01G000560.1	402	45595.37	9.33	61.28	65.42	−0.994	Ghir_D01	396637–399307
GhHsp40-81	Ghir_D06G003390.1	456	50631.9	6.8	40.93	65.9	−0.516	Ghir_D06	4035348–4042307
GhHsp40-82	Ghir_A06G011640.2	521	55679.54	8.75	37.66	67.2	−0.425	Ghir_A06	55723023–55731818
GhHsp40-83	Ghir_D06G003390.2	447	49554.69	6.8	41.26	65.48	−0.519	Ghir_D06	4035348–4042307
GhHsp40-84	Ghir_D06G013050.1	507	54128.89	8.88	36.92	68.28	−0.392	Ghir_D06	37095612–37104427
GhHsp40-85	Ghir_D11G011810.4	344	38756.14	6.19	38.43	81.05	−0.507	Ghir_D11	10629342–10634402
GhHsp40-86	Ghir_D12G001560.2	435	47429.13	9.31	36.66	79.31	−0.388	Ghir_D12	1877205–1880794
GhHsp40-87	Ghir_D11G011810.3	370	41862.84	6.57	39.12	80.59	−0.444	Ghir_D11	10611312–10616648
GhHsp40-88	Ghir_D05G016230.1	344	37960.17	9.14	37.57	66.92	−0.667	Ghir_D05	14173304–14175939
GhHsp40-89	Ghir_D05G029280.1	337	37344.31	5.97	37.7	63.62	−0.655	Ghir_D05	29413842–29419184
GhHsp40-90	Ghir_D05G029280.2	337	37344.31	5.97	37.7	63.62	−0.655	Ghir_D05	29413842–29419184
GhHsp40-91	Ghir_D05G029280.3	336	37287.26	5.97	37.42	63.81	−0.656	Ghir_D05	29415300–29419026
GhHsp40-92	Ghir_D09G010620.1	417	46231.23	5.68	38.59	64.53	−0.729	Ghir_D09	37384437–37386519
GhHsp40-93	Ghir_A08G005820.1	444	48375.84	9.04	36.09	73.49	−0.44	Ghir_A08	7358381–7365237

**TABLE 2:** Information of *GhHsp70* gene family in cotton.

Gene name	Accession number	Number of amino acid	Molecular weight	Theoretical PI	Instability index	Aliphatic index	Grand average of hydropathicity	Chr	Position
GhHsp70-1	Ghir_D03G009660.1	644	71209.64	5.04	30.88	87.34	−0.489	Ghir_D03	33,779,314–33,782,987
GhHsp70-2	Ghir_D11G022150.1	682	73337.02	5.78	41.43	87.29	−0.317	Ghir_D11	30,577,512–30582,084
GhHsp70-3	Ghir_D01G023190.1	512	56076.86	5.59	30.54	87.97	−0.25	Ghir_D01	62,526,089–62,529,649
GhHsp70-4	Ghir_D09G008770.1	827	91780.68	5.35	41.81	79.15	−0.426	Ghir_D09	34,599,685–34,606,758
GhHsp70-5	Ghir_A09G022410.1	589	63160.86	5.64	28.8	92.48	−0.155	Ghir_A09	78,649,508–78,653,668
GhHsp70-6	Ghir_D06G018900.1	648	71010.52	5.1	33.72	82.65	−0.412	Ghir_D06	60,526,788–60,530,581
GhHsp70-7	Ghir_A09G022410.2	706	75722.48	5.25	29.67	85.59	−0.335	Ghir_A09	78,650,175–78,653,668
GhHsp70-8	Ghir_D12G001770.1	677	72377.96	5.62	33.51	85.33	−0.315	Ghir_D12	2,142,220–2146,019
GhHsp70-9	Ghir_D09G016060.1	646	70871.35	5.1	31.71	83.36	−0.393	Ghir_D09	44,438,587–44,441,708
GhHsp70-10	Ghir_D11G036090.1	647	70982.51	5.14	35.26	82.16	−0.416	Ghir_D11	72,641,367–72,646,283
GhHsp70-11	Ghir_D06G019840.1	886	98521.15	5.58	36.63	83.1	−0.521	Ghir_D06	62,218,829–62,226,284
GhHsp70-12	Ghir_D03G009660.2	548	60583.54	5.01	30.64	85.22	−0.544	Ghir_D03	33,779,379–33,782,987
GhHsp70-13	Ghir_D09G022210.1	567	62652.27	5	39.86	104.29	−0.007	Ghir_D09	50,185,447–50187,150
GhHsp70-14	Ghir_D08G012060.1	666	73252.07	5.2	28.92	88.72	−0.427	Ghir_D08	41,269,249–41,272,907
GhHsp70-15	Ghir_A09G009090.1	178	20494.04	4.98	52.12	64.1	−0.661	Ghir_A09	59,566,118–59,571,395
GhHsp70-16	Ghir_D10G007540.2	797	88106.23	5.53	43.94	78.49	−0.486	Ghir_D10	7,941,452–7947,444
GhHsp70-17	Ghir_D10G007540.1	854	94124.03	5.51	43.53	78.62	−0.447	Ghir_D10	7,941,332–7948,057
GhHsp70-18	Ghir_A10G000890.2	774	86938.36	5.47	49.39	87.95	−0.367	Ghir_A10	6,82,865–6,88,775
GhHsp70-19	Ghir_A12G011800.1	646	70758.15	5.22	30.79	84.1	−0.422	Ghir_A12	79,638,721–79,640,661
GhHsp70-20	Ghir_A10G000890.1	774	86938.36	5.47	49.39	87.95	−0.367	Ghir_A10	682,283–688775
GhHsp70-21	Ghir_A10G014720.1	62	6794.58	4.34	14.66	69.35	−0.363	Ghir_A10	79,013,102–79,014,299
GhHsp70-22	Ghir_A03G004890.1	648	70838.23	5.25	33.88	79.04	−0.435	Ghir_A03	8,235,559–8238,853
GhHsp70-23	Ghir_D05G009590.1	650	71173.67	5.13	34.03	80.14	−0.438	Ghir_D05	7,975,993–7,981,588
GhHsp70-24	Ghir_A02G000840.2	704	75430.18	5.24	31.27	86.39	−0.329	Ghir_A02	612,113–6,16,280
GhHsp70-25	Ghir_A02G000840.1	704	75430.18	5.24	31.27	86.39	−0.329	Ghir_A02	612,120–6,16,828
GhHsp70-26	Ghir_D09G008770.2	856	94596.59	5.32	42.89	77.28	−0.46	Ghir_D09	34,599,685–34,606,758
GhHsp70-27	Ghir_A06G018820.1	667	74628.53	5.35	33.86	75.58	−0.719	Ghir_A06	119,251,889–119,259,372
GhHsp70-28	Ghir_A08G011300.1	666	73238.04	5.2	28.63	88.57	−0.427	Ghir_A08	74,995,710–74,999,519
GhHsp70-29	Ghir_D13G012000.1	656	72737.55	5.19	29.27	89.62	−0.439	Ghir_D13	37,130,760–37,133,507
GhHsp70-30	Ghir_A01G021670.1	648	71215.64	5.07	36.9	82.02	−0.427	Ghir_A01	116,946,783–116,949,869
GhHsp70-31	Ghir_D12G001780.1	677	72426.05	5.71	33.76	85.48	−0.308	Ghir_D12	2,156,616–2161,885
GhHsp70-32	Ghir_A10G022120.1	652	71221.69	5.16	34.74	82.75	−0.401	Ghir_A10	110,661,155–110,663,923
GhHsp70-33	Ghir_A10G019660.1	879	98486.02	5.61	35.66	83.67	−0.447	Ghir_A10	104,784,896–104,794,468
GhHsp70-34	Ghir_A01G021680.1	648	71215.64	5.07	36.9	82.02	−0.427	Ghir_A01	116,950,453–116,954,134
GhHsp70-35	Ghir_D03G013890.1	648	70924.28	5.21	34.99	78.89	−0.444	Ghir_D03	45,240,594–45,243,935
GhHsp70-36	Ghir_A11G022260.2	484	52303.92	8.79	38.13	93.72	−0.111	Ghir_A11	57,589,530–57,593,175
GhHsp70-37	Ghir_A11G022260.1	678	72971.5	5.59	42.81	87.08	−0.323	Ghir_A11	57,589,467–57,593,175
GhHsp70-38	Ghir_D06G018900.2	614	67116.11	5	35.23	83.73	−0.361	Ghir_D06	60526840–60529,321
GhHsp70-39	Ghir_D10G012820.1	706	75707.5	5.34	31.11	86.42	−0.349	Ghir_D10	20,867,072–20870,660
GhHsp70-40	Ghir_D02G000840.1	704	75441.24	5.21	31.41	86.66	−0.335	Ghir_D02	649,389–653670
GhHsp70-41	Ghir_A10G007680.1	855	94337.25	5.36	42.7	78.06	−0.442	Ghir_A10	12,352,269–12,365,665
GhHsp70-42	Ghir_A06G018020.1	648	70996.5	5.1	34.53	82.65	−0.409	Ghir_A06	117,617,701–117,620,932
GhHsp70-43	Ghir_A02G010300.1	666	73381.2	5.13	29.05	87.55	−0.44	Ghir_A02	43,500,572–43,504,275
GhHsp70-44	Ghir_A09G022990.1	550	60684.81	4.95	41.72	103.07	0.02	Ghir_A09	79,157,921–79,159,673
GhHsp70-45	Ghir_A11G001960.1	667	73533.45	5.17	27.92	87.12	−0.443	Ghir_A11	1,692,865–1698,001
GhHsp70-46	Ghir_A11G001960.2	549	60721.73	5.04	29.98	84.17	−0.551	Ghir_A11	1,692,995–1698,001
GhHsp70-47	Ghir_D11G002010.1	667	73465.43	5.13	28.55	87.57	−0.427	Ghir_D11	1,666,656–1670,686
GhHsp70-48	Ghir_D08G002660.5	757	84871.81	6	49.64	81.96	−0.394	Ghir_D08	2,355,898–2360,423
GhHsp70-49	Ghir_D08G002660.4	757	84871.81	6	49.64	81.96	−0.394	Ghir_D08	2,355,898–2360,423
GhHsp70-50	Ghir_A08G002560.1	708	79689.96	6.15	40.87	81.99	−0.417	Ghir_A08	2,439,155–2443,466
GhHsp70-51	Ghir_D08G002660.6	757	84871.81	6	49.64	81.96	−0.394	Ghir_D08	2,355,898–2360,423
GhHsp70-52	Ghir_A05G009880.1	650	71112.63	5.13	34.33	80.74	−0.433	Ghir_A05	8,941,782–8944,916
GhHsp70-53	Ghir_D08G002660.1	757	84871.81	6	49.64	81.96	−0.394	Ghir_D08	2,355,882–2360,423
GhHsp70-54	Ghir_D09G021670.2	706	75625.32	5.17	30.18	85.47	−0.338	Ghir_D09	49,724,683–49,728,806
GhHsp70-55	Ghir_D09G021670.1	500	54067.76	4.83	30.93	95.92	−0.202	Ghir_D09	49,724,618–49,728,806
GhHsp70-56	Ghir_D10G021370.1	879	98686.07	5.51	35.92	83.46	−0.473	Ghir_D10	58,083,962–58,093,460
GhHsp70-57	Ghir_A11G035220.1	647	70808.29	5.14	35.95	82.02	−0.402	Ghir_A11	122,939,287–122,942,256
GhHsp70-58	Ghir_D08G002660.3	757	84871.81	6	49.64	81.96	−0.394	Ghir_D08	2,355,898–2360,423
GhHsp70-59	Ghir_D08G002660.2	757	84871.81	6	49.64	81.96	−0.394	Ghir_D08	2,355,898–2360,423
GhHsp70-60	Ghir_D09G022190.1	570	61992.54	5.55	36.36	100.37	0.069	Ghir_D09	50,176,520–50179,878
GhHsp70-61	Ghir_D11G001970.1	667	73532.42	5.11	29.38	87.27	−0.441	Ghir_D11	1,642,807–1648,189
GhHsp70-62	Ghir_D11G001970.2	667	73532.42	5.11	29.38	87.27	−0.441	Ghir_D11	1,642,807–1646,917
GhHsp70-63	Ghir_A13G011430.1	656	72773.58	5.22	32.45	89.18	−0.459	Ghir_A13	73,776,809–73,779,640
GhHsp70-64	Ghir_D03G017540.1	648	70807.31	5.26	34.21	84.75	−0.4	Ghir_D03	50,603,565–50605,943
GhHsp70-65	Ghir_A11G002000.1	667	73532.47	5.17	27.41	87.42	−0.439	Ghir_A11	1,727,873–1732,029
GhHsp70-66	Ghir_A09G022980.1	570	61935.41	5.55	36.85	99.18	0.055	Ghir_A09	79142806–79,145,981
GhHsp70-67	Ghir_A09G016580.1	646	70869.38	5.1	31.96	83.67	−0.389	Ghir_A09	72,720,394–72,723,871
GhHsp70-68	Ghir_D12G011700.1	503	55049.76	5.85	32	89.2	−0.261	Ghir_D12	39,937,391–39,938,940
GhHsp70-69	Ghir_D10G001670.1	774	86800.17	5.38	47.55	88.95	−0.346	Ghir_D10	1,371,576–1377,781
GhHsp70-70	Ghir_D10G001670.2	774	86800.17	5.38	47.55	88.95	−0.346	Ghir_D10	1,372,045–1377,830
GhHsp70-71	Ghir_D10G001670.5	774	86800.17	5.38	47.55	88.95	−0.346	Ghir_D10	1,372,300–1377,781
GhHsp70-72	Ghir_D10G001670.3	744	83642.85	5.56	45.8	90.19	−0.322	Ghir_D10	1,372,300–1377,822
GhHsp70-73	Ghir_A13G024720.1	652	71383.89	5.15	34.38	82.88	−0.424	Ghir_A13	108,237,760–108,240,420
GhHsp70-74	Ghir_D10G001670.4	774	86800.17	5.38	47.55	88.95	−0.346	Ghir_D10	1,372,300–1377,803
GhHsp70-75	Ghir_D10G024170.1	652	71337.72	5.13	36.98	82.15	−0.412	Ghir_D10	63,330,563–63,333,307
GhHsp70-76	Ghir_A12G001770.1	676	72230.79	5.62	33.76	85.33	−0.31	Ghir_A12	2,329,085–2332,572
GhHsp70-77	Ghir_D13G025490.1	652	71383.89	5.15	34.38	82.88	−0.424	Ghir_D13	63,196,915–63,199,537

### *Chromosomal* location analysis of *Hsp40s* and *Hsp70s* in cotton species

**FIGURE 1:**
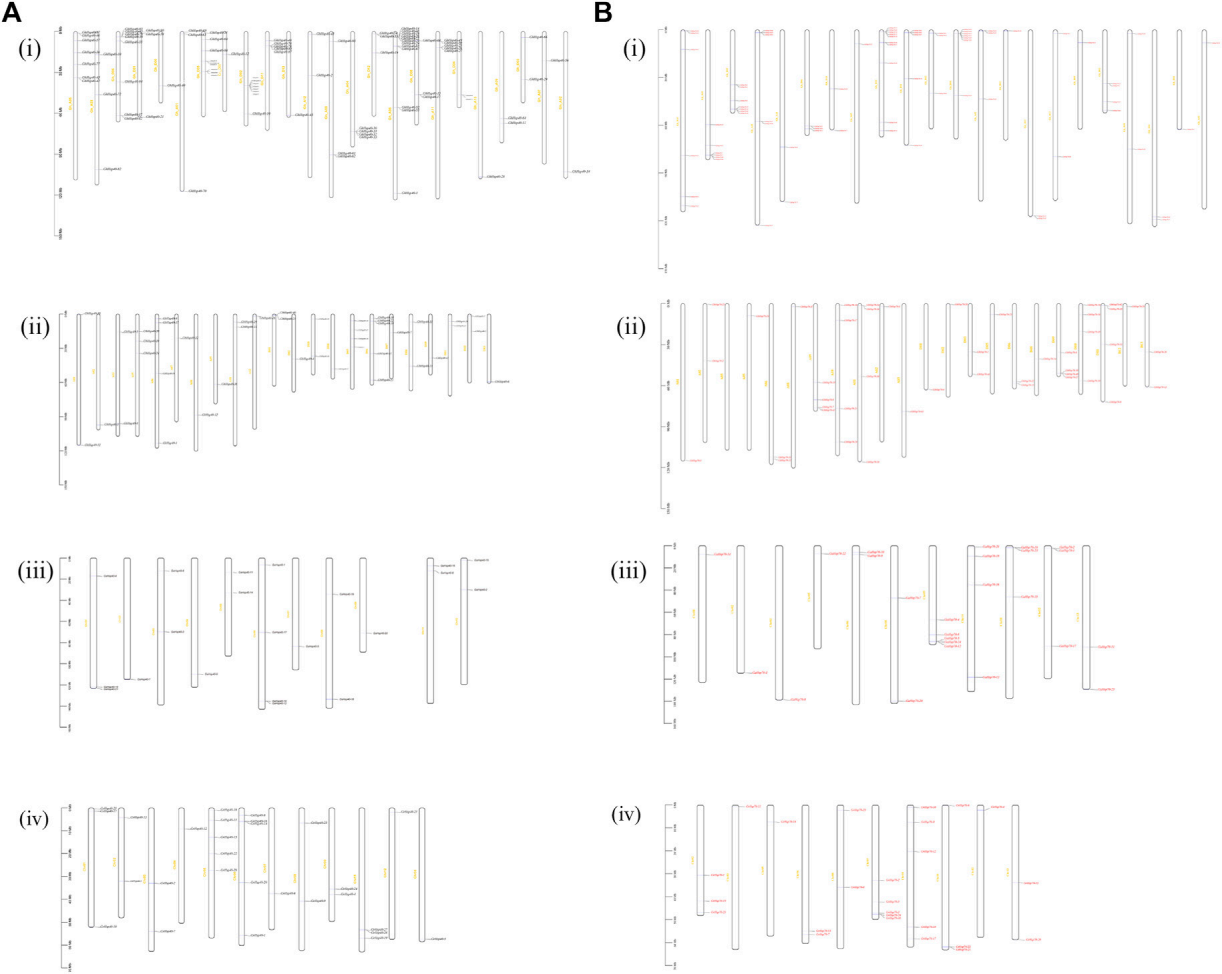
Chromosomal localization analysis of *Hsp40s* and *Hsp70s* gene family members in four cotton species. **(A)**: (i) *Hsp40s* gene family members in *G. hirsutum* (Gh), (ii) *Hsp40s* gene family members in *G. barbadense* (Gb), (iii) *Hsp40s* gene family members in *G. arboreum* (Ga), (iv) *Hsp40s* gene family members in *G. raimondii* (Gr). **(B)**: (i) *Hsp70s* gene family members in *G. hirsutum* (Gh), (ii) *Hsp70s* gene family members in *G. barbadense* (Gb), (iii) *Hsp70s* gene family members in *G. arboreum* (Ga), (iv) *Hsp70s* gene family members in *G. raimondii* (Gr).

According to the GFF files of *G. hirsutum* (NDM8), *G. raimondii* (JGI_v2.1), *G. arboreum* (CRI_v3.0), *G. barbadense* (HAU v1), the online software MG2C conducted a visual analysis of its location (Figure 1). The results show that 93 *GhHsp40s* are evenly distributed in 12 At_subgenomes and 12_Dt subgenomes (Figure 1A), of which 39 *GhHsp40s* are in the At_subgenome, and 12 are in the Chr06 chromosome (*GhHsp40-1*, *GhHsp40-14*, *GhHsp40-16*, *GhHsp40-18*, *GhHsp40-20*, *GhHsp40-22*, *GhHsp40-23*, *GhHsp40-24*, *GhHsp40-52*, *GhHsp40-53*, *GhHsp40-65*, *GhHsp40-67*). There were 54 *GhHsp40s* in the Dt subgenome, and more *GhHsp40s* (8 *GhHsp40s*) were distributed in the Chr06 chromosome. Ninety-one *GbHsp40s* were evenly distributed in 10 At_subgenomes and 12 Dt_subgenomes, and 1-5 *GbHsp40s* were distributed on each chromosome. The distribution of *GaHsp40s* gene on chromosome is similar to that of *GhHsp40s* in At_subgenome. At the same time, the distribution of *GrHsp40s* gene on chromosomes is also similar to that of *GhHsp40s* in At_subgenome, while on Chr06 chromosome, there is an additional *Hsp40s-8* distributed on *GrHsp40s*, with less *Hsp40-52*, *Hsp40-53*, *Hsp40-65*, and *Hsp40-67*. This indicates that there is gene deletion or duplication in the genome of upland cotton.

Seventy-seven *GhHsp70s* were uniformly distributed in 11 At_subgenomes and 11 Dt_subgenomes (Figure 1B), among which 32 *GhHsp70s* were found in At_subgenomes, and most of the chromosomes had 2-3 *GhHsp70s*. The number of chromosomes distributed on Chr09, Chr10 and Chr11 was 6 *GhHsp70s*. There were 45 *GhHsp70s* in Dt_subgenome, and there were 10 GhHsp70s in Chr10 chromosome, which were *GhHsp70-16*, *GhHsp70-17*, *GhHsp70-39*, *GhHsp70-56*, *GhHsp70-69*, *GhHsp70-70*, *GhHsp70-71*, *GhHsp70-72*, *GhHsp70-74*, *GhHsp70-75*. 44 *GbHsp70s* were evenly distributed in 11 At_subgenomes and 11 Dt_subgenomes, with 21 and 23 *GbHsp70s*, respectively. The 25 *GaHsp70s* were similar to those of *GhHsp70s* in the Dt_subgenome. For example, *Hsp70-4* and *Hsp70-6* were found in both genomes at similar locations on Chr09. The distribution of 25 *GrHsp70s* was similar to that of *GhHsp70s* in the At_subgenome, for example, *GrHsp70s-18* in the upper and lower ends of the Chr09 chromosome and *GhHsp70s-18* in the upper end of the Chr09 chromosome in the At_subgenome, indicating an inversion in the chromosomes of these cotton species. In addition, it was also found that Hsps were conservatively distributed on Chr05, Chr06, Chr09, Chr10, Chr11 and Chr13 of four cotton species.

### *Phylogenetic* analysis of Hsp40s and Hsp70s

**FIGURE 2:**
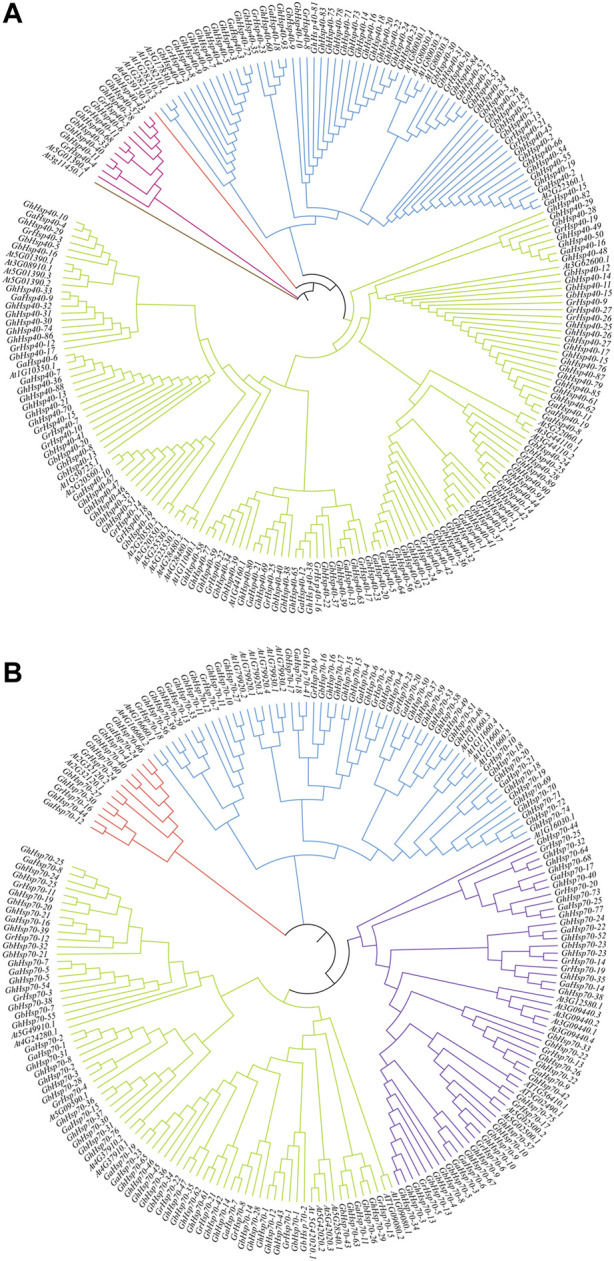
Phylogenetic tree of *Hsp40s* and *Hsp70s* gene family members from 5 species. **(A)**: *Hsp40s* gene family members in 5 species. **(B)**: *Hsp70s* gene family members in 5 species. The gene sequences were selected from *G. hirsutum* (Gh), *G. arboreum* (Ga), *G. barbadense* (Gb), *G. raimondii* (Gr), *A. thaliana* (At).

In order to clarify the evolutionary relationship between *Hsp40s* and *Hsp70s*, we imported the full-length protein sequences of 291 *Hsp40s,* 171 *Hsp70s*, *AtHsp40s* and *AtHsp70s* into MEGA7.0 software for comparison. The phylogenetic tree was constructed using the adjacency method, and the *Hsp40s/Hsp70* subgroups of *G. hirsutum*, *G. raimondii*, *G. arboreum*, *G. barbadense* and *Arabidopsis thaliana* were classified according to the number of branches in the phylogenetic tree (Figure 2). Phylogenetic analysis of *Hsp40s* family members (Figure 2A) revealed that two species specific *Hsp40s* branches appeared (*At3G11451.1* and *At4G39150.3*), and all the remaining five species of *Hsp40s* could be classified into three subgroups. They were named as Hsp40s-I, II and III subgroups. The Hsp40s-I subgroup had the least number of *Hsp40s*, including 6 *GhHsp40s*, 3 *GbHsp40s*, 2 *GaHsp40s*, and 1 *AtHsp40s*, 12 *Hsp40s* in total. Hsp40s-II subgroup includes 33 *GhHsp40s*, 11 *GbHsp40s*, 5 *GaHsp40s*, 6 *GrHsp40s*, 9 *AtHsp40s*, and 64 *Hsp40s* members in total. The Hsp40s-III subgroup has the largest number of members, including 138 *GhHsp40s*, 29 *GbHsp40s*, 18 *GrHsp40s*, 15 *GrHsp40s*, and 19 *AtHsp40s*, totaling 213 *Hsp40s*.

The phylogenetic analysis of members of the Hsp70s gene family shows that (Figure 2B), the *Hsp70s* gene family was different from the *Hsp40s* gene family, and there was no species specific *Hsp70s* branch. All *Hsp70s* of the five species could be grouped into four subgroups named Hsp70s-I, II, III, and IV. Similar to the *Hsp40s* gene family, the Hsp70s-I subgroup had the smallest number of Hsp70s members, with only 13 *Hsp70s* members, including 4 *GhHsp70s*, 3 *GbHsp70s*, 2 *GaHsp70s*, 2 *GrHsp70s*, and 2 *AtHsp70s*. There was no significant difference in the number of members of Hsp70s-II and III subgroups, and the distribution of the number of five species in the two subgroups was similar. There were 59 *Hsp70s* in the Hsp70-II subgroup, including 24 *GhHsp40s*, 11 *GbHsp70s*, 6 *GaHsp70s*, 6 *GrHsp70s*, and 12 *AtHsp70s*. There were 57 *Hsp70s* in the Hsp70-III subgroup, including 21 *GhHsp70s*, 11 *GbHsp70s*, 7 *GaHsp70s*, 8 *GrHsp70s*, and 10 *AtHsp70s*. Hsp70s-IV is the subgroup with the largest distribution of *Hsp70s* gene family members. There are 77 *Hsp70s* members, including 26 *GhHsp70*s, 19 *GbHsp70s*, 10 *GaHsp70s*, 9 *GrHsp70s* and 11 *AtHsp70s*.

The homologous genes of each species mainly contain duplicate genes. The phylogenetic analysis shows that there are specific *Hsp40s* and *Hsp70s* gene duplication events in four cotton species. In the *Hsp40s* gene family, we identified 8 pairs of homologous genes (*Hsp40-1*, *2*, *3*, *4*, *11*, *15*, *26*, *27*). Fourteen pairs of homologous genes were identified in the *Hsp70s* gene family (*Hsp70-1*, *2*, *5*, *6*, *10*, *13*, *14*, *15*, *16*, *18*, *21*, *23*, *24*, *25*).

### *Protein* features of Hsp40s and Hsp70s

**FIGURE 3:**
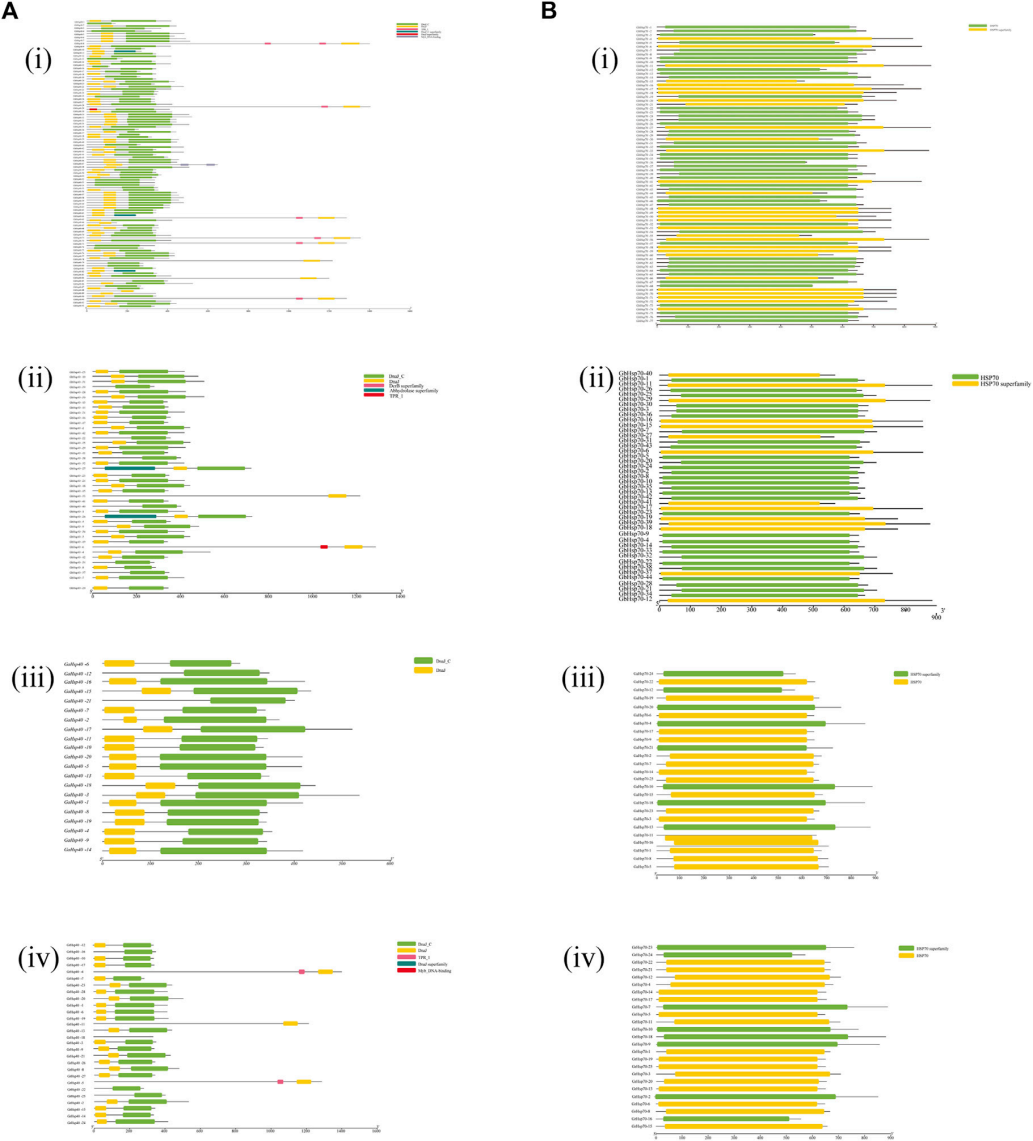
Protein features of Hsp40s and Hsp70s in four cotton species. **(A)**: (i) Hsp40s protein in *G. hirsutum* (Gh), (ii) Hsp40s protein in *G. barbadense* (Gb), (iii) Hsp40s protein in *G. arboreum* (Ga), (iv) Hsp40s protein in *G. raimondii* (Gr). **(B)**: (i) Hsp70s protein in *G. hirsutum* (Gh), (ii) Hsp70s protein in *G. barbadense* (Gb), (iii) Hsp70s protein in *G. arboreum* (Ga), (iv) Hsp70s protein in *G. raimondii* (Gr).

**FIGURE 4:**
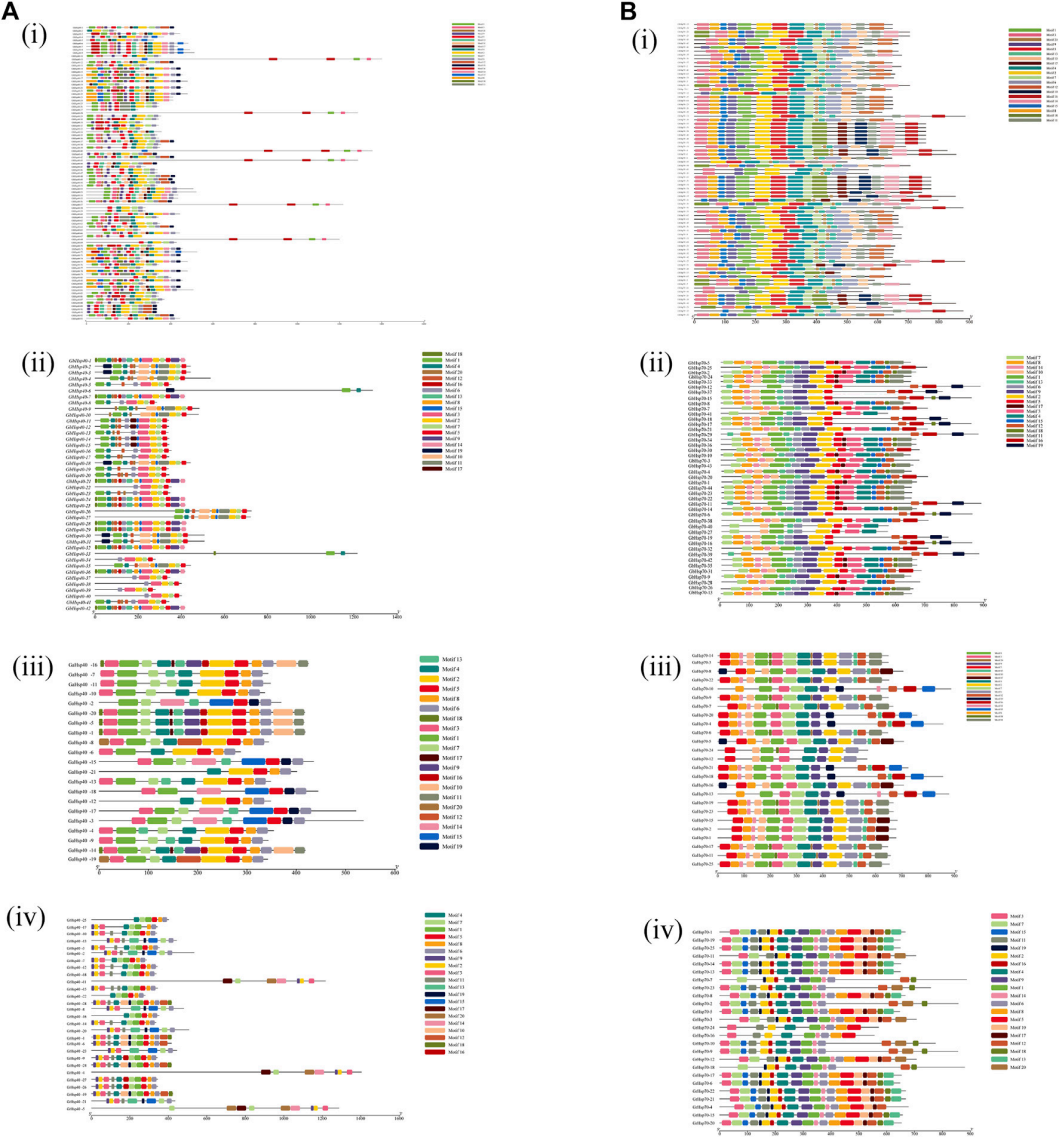
The motifs of Hsp40s and Hsp70s sequences from our cotton species. **(A)**: (i) Hsp40s protein in *G. hirsutum* (Gh), (ii) Hsp40s protein in *G. barbadense* (Gb), (iii) Hsp40s protein in *G. arboreum* (Ga), (iv) Hsp40s protein in *G. raimondii* (Gr). **(B)**: (i) Hsp70s protein in *G. hirsutum* (Gh), (ii) Hsp70s protein in *G. barbadense* (Gb), (iii) Hsp70s protein in *G. arboreum* (Ga), (iv) Hsp70s protein in *G. raimondii* (Gr).

In order to identify the conserved domains of Hsp40s and Hsp70s, we submitted 291 Hsp40s and 171 Hsp70s protein sequences to the CDD tool of NCBI for prediction (Figure 3). Hsp40s protein contains eight conservative domains (Figure 3A): DnaJ, DnaJ_ C, TPR_ I, DnaJ_ C superfamily, DnaJ superfamily, MYB DNA-binding, DcrB superfamily, abhydrolase superfamily. The Hsp40s of the four cotton species all have conserved DnaJ and DnaJ_C domains, which are consistent with the Hsp40s of other species. In particular, GaHsp40s is different from the other three cotton species in that it has only DnaJ and DnaJ_C domains. These results indicate that Hsp40s has increased and lost some conserved domains during evolution. Notably, only *GaHsp40s* contain two domains (DnaJ and DnaJ_C domains) (Figure 3A iii), suggesting that they are the most conserved. GhHsp40s and GrHsp40s have DnaJ_ C superfamily, DnaJ superfamily, MYB_ DNA-binding, and MYB_ DNA-binding domains. While *GrHsp40s* has special DcrB superfamily and abhydrolase superfamily domains, which indicates that they are located in G Some special functions may be obtained in *G. hirsutum*, *G. raimondii*, *G. barbadense*. The *Hsp70s* proteins in four cotton species all contain only two conservative domains (Figure 3B): HSP70, HSP70 superfamily. These results indicate that these two domains of Hsp70s protein are very conservative during evolution. In addition, we used MEME online software to analyze the motif characteristics of Hsp40s and Hsp70s, and identified 20 conserved motifs in *Hsp40s* and *Hsp70s* gene families (Figure 4).

### Collinearity *analysis Hsp40s* and *Hsp70s*

**FIGURE 5:**
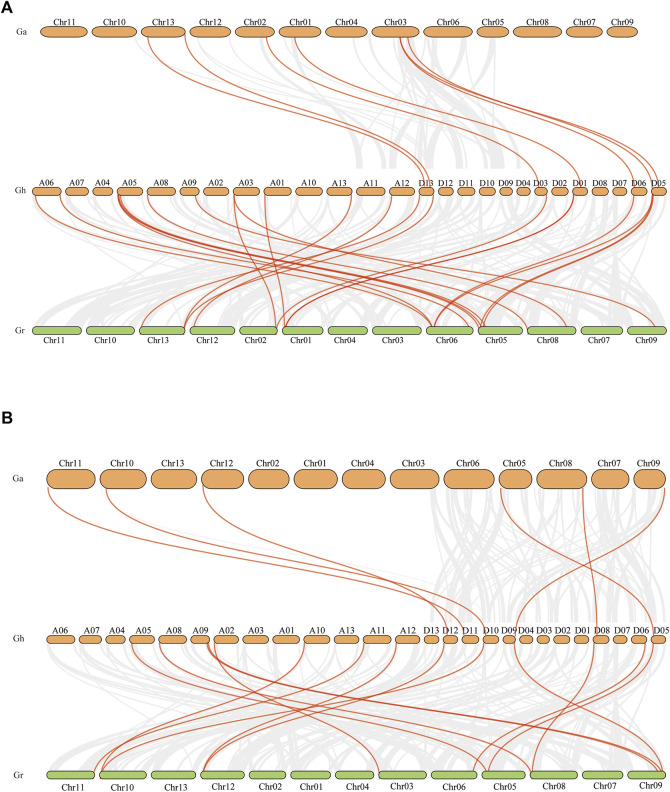
Collinearity analysis of *Hsp40s* and *Hsp70s* genes. **(A)**: Collinearity analysis of *Hsp40s* genes in *G. hirsutum*, *G. arboreum*, and *G. raimondii*; **(B)**: Collinearity analysis of *Hsp70s* genes in *G. barbadense*, *G. arboreum*, and *G. raimondii.*

Tandem and fragment DNA replication are the main driving forces driving the expansion of gene families and the evolution of the entire genome. In order to explore the evolutionary relationship between *Hsp40s* and *Hsp70s* of diploid and tetraploid cotton, we analyzed the collinearity between diploid and tetraploid cotton. The results showed that six *GaHsp40s* were collinear with six *GhHsp40s* genes. There was a collinear relationship between *GhHsp40s* of 16 *G. hirsutum* and *GbHsp40s* of 12 *G. raimondii* (Figure 5A). Six *GaHsp70s* genes of *G. arboreum* were collinear with six *GhHsp70s* of *G. hirsutum*. Fourteen *GhHsp70s* genes of *G. hirsutum* were collinear with eight *GbHsp70s* of *G. raimondii* (Figure 5B). Collinearity analysis showed that each Hsp40 or Hsp70 from diploid cotton (*G. arboreum* or *G. raimondii*) corresponded to at least one homologous gene in the At_and Dt_subgenomes of tetraploid cotton species (G. hirsutum or G. marimondii).

### *Cis*-promoter *element* analysis of *Hsp40s* and *Hsp70s*

**FIGURE 6:**
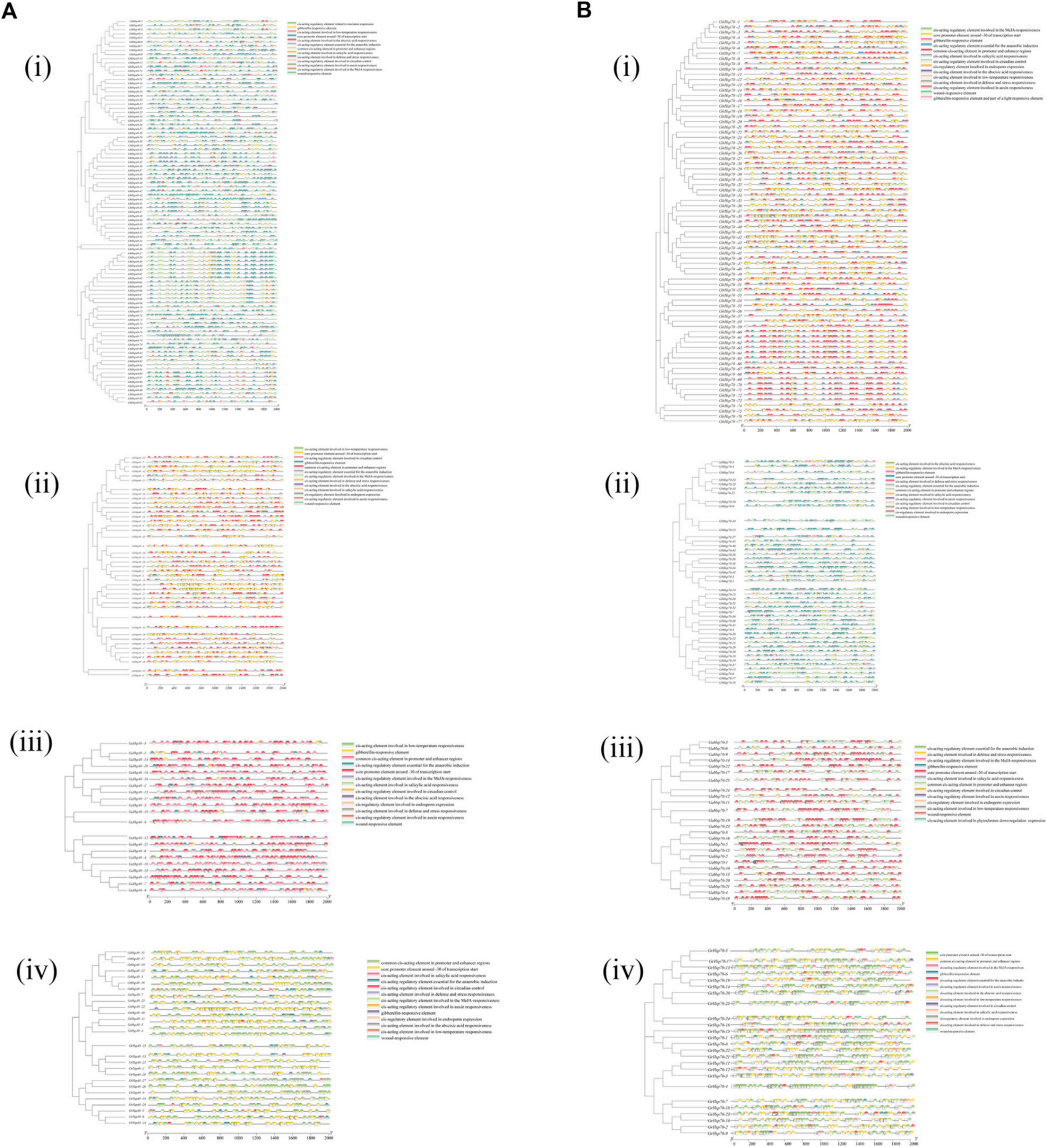
The distribution of cis-acting elements in upstream sequence of *Hsp40s* and *Hsp70s* in four cotton species. **(A)**: (i) *Hsp40s* gene family members in *G. hirsutum* (Gh), (ii) *Hsp40s* gene family members in *G. barbadense* (Gb), (iii) *Hsp40s* gene family members in *G. arboreum* (Ga), (iv) *Hsp40s* gene family members in *G. raimondii* (Gr). **(B)**: (i) *Hsp70s* gene family members in *G. hirsutum* (Gh), (ii) *Hsp70s* gene family members in *G. barbadense* (Gb), (iii) *Hsp70s* gene family members in *G. arboreum* (Ga), (iv) *Hsp70s* gene family members in *G. raimondii* (Gr).

We further analyzed the *cis*-regulatory elements in the upstream region of *Hsp40s* and *Hsp70s*. The analysis results showed that there were 13 different cis-acting elements in Hsp40s of the four cotton seeds (Figure 6A), which were divided into two types according to their different functions, namely, the assumed abiotic stress response elements and the hormone response elements. In *GhHsp40s* promoter, hormone-related elements occupy the largest proportion. Examples include gibberellin-responsive element, salicylic acid responsive element and abscisic acid responsive element. The second is low temperature responsive element, anaerobic induction responsive element and wound-responsive element. Except that the promoter of *GhHsp40s* contains one hormone response element, the promoters of other *GhHsp40s* all have at least two hormone response elements. Among the promoters of *GbHsp40s*, defense and stress responsive elements account for the largest proportion, followed by gibberellin responsive element and MeJA responsive element. Among *GaHsp40s* promoters, low temperature responsive element, anaerobic induction responsive element and wow responsive element account for the most. Among the promoters of *GrHsp40s*, the ratio of defense and stress responsive element, wound responsive element and gibberellin responsive element is the highest.

We identified 15 distinct cis-acting elements in *GhHsp70s* promoters of four cotton species (Figure 6B), which were classified into three categories based on their roles, including photoresponsive, hormonal, and abiotic stress response elements. In the promoter of *GhHsp70s*, salicylic acid responsive element, anaerobic induction responsive element and wound-responsive element. Among the promoters of *GbHsp70s*, abscisic acid responsive element and anaerobic induction responsive element make up the largest proportion. Second is the MeJA responsive element. Among *GaHsp70s* promoters, we found that the phytochrome downregulation expression element, which is not found in the *Hsp70s* promoter, and low temperature responsive element, defense and stress responsive element and anaerobic induction responsive element, which are non-biological stress related elements, account for the largest proportion, followed by the hormone related element gibberellin responsive element. For *GrHsp70s* promoter, anaerobic induction responsive element occupies the largest proportion, followed by hormone-related element gibberellin-responsive element.

### Expression analysis of *GhHsp40s* and *GhHsp70s* genes under biological and abiotic stress conditions

**FIGURE 7:**
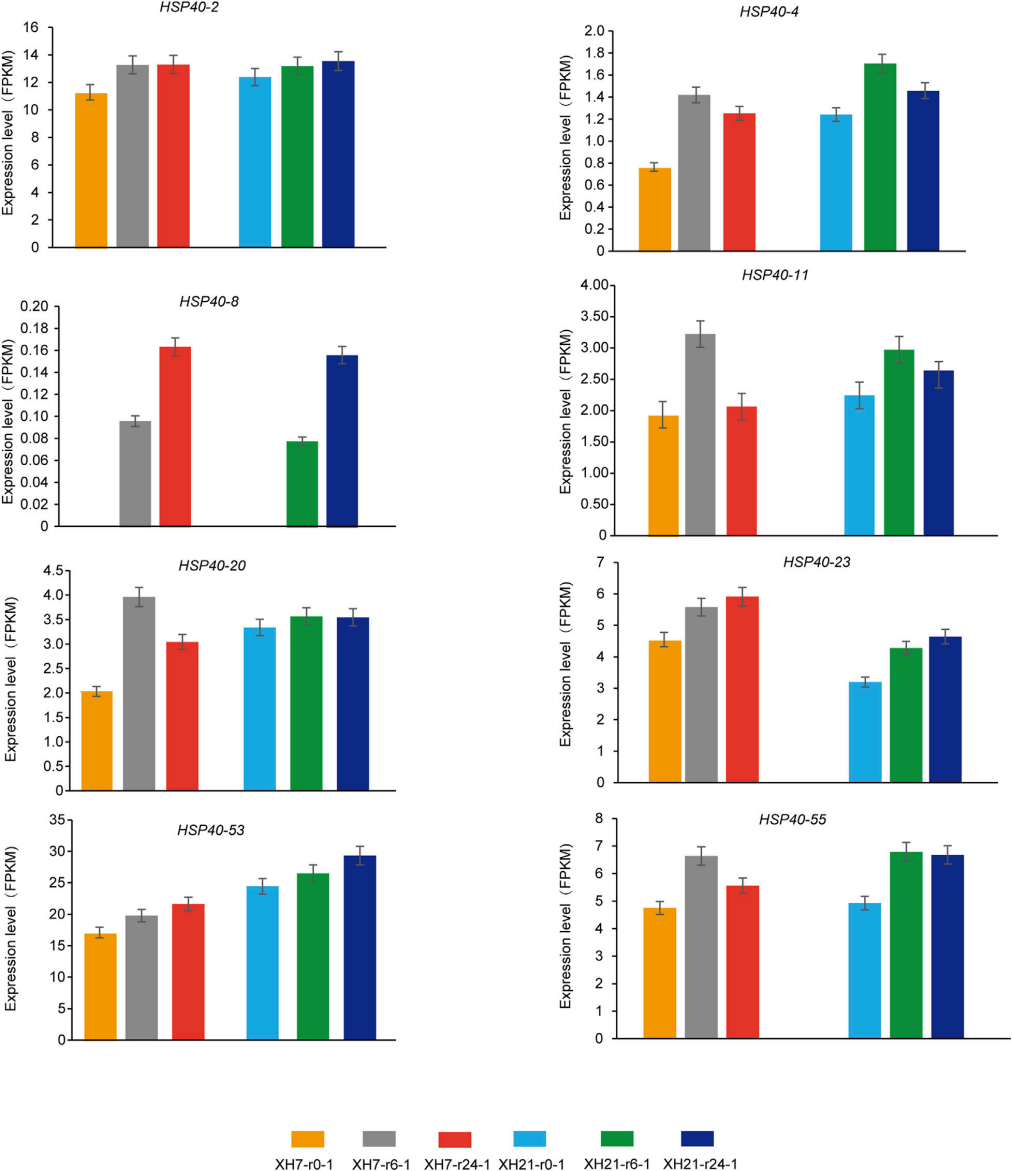
Temporal and spatial expression profiles of *Hsp40s* gene family members after inoculation with *V. dahliae* infection.

**FIGURE 8:**
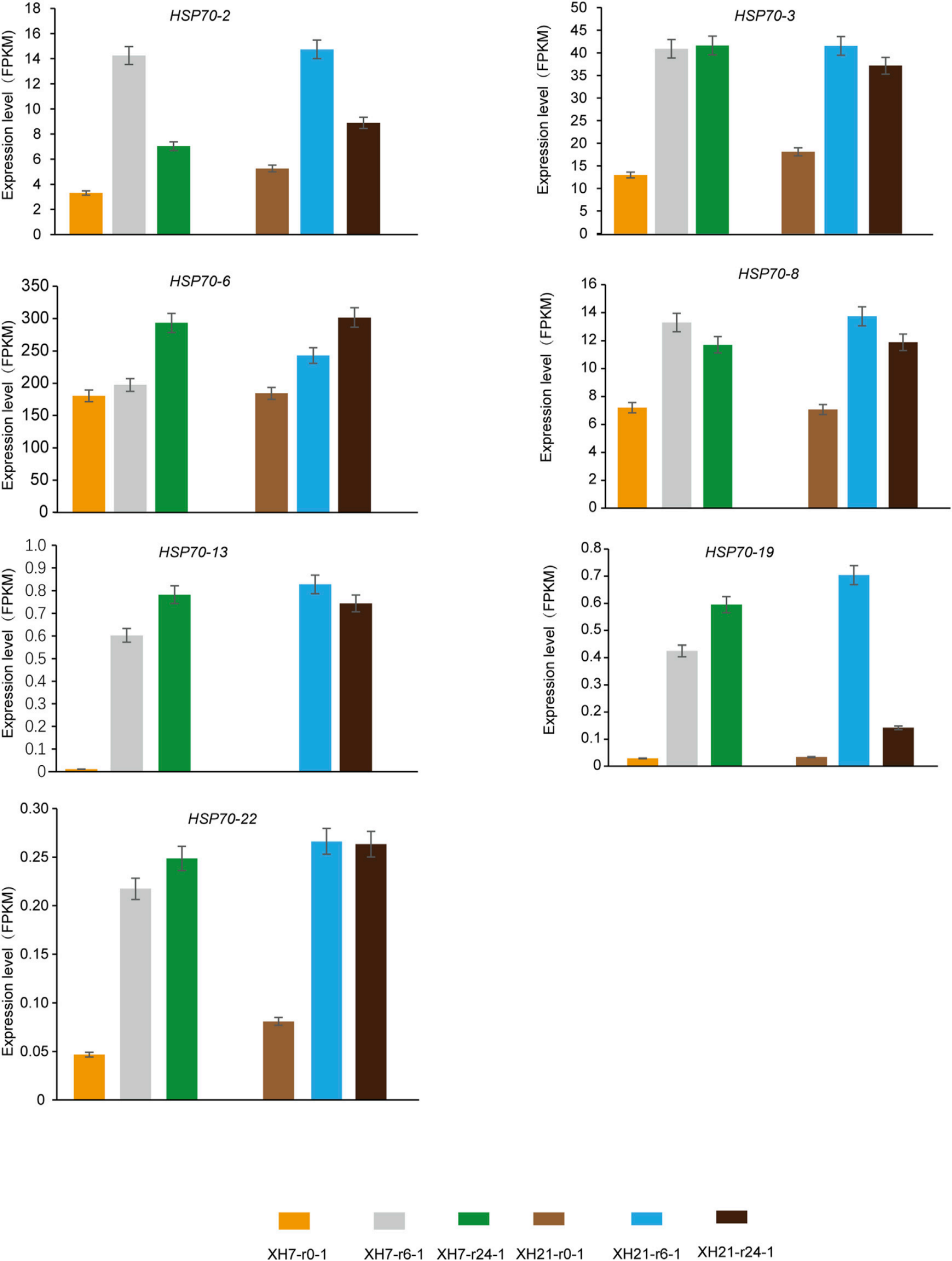
Temporal and spatial expression profiles of *Hsp70s* gene family members after inoculation with *V. dahliae* infection.

Upland cotton is one of the important economic crops in the world. At present, it dominates the world cotton trade and natural cotton linter (Li et al., 2015; Hu et al., 2019). In order to further understand the potential function of *GhHsp40s* and *GhHsp70s* involved in the response to *V. dahliae* and abiotic stress (high temperature, low temperature, salt, and drought), we analyzed the profile of all members of *GhHsp40s* and *GhHsp70s* gene families. We found that, compared to the control group, *GhHsp40-2*, *GhHsp40-4*, *GhHsp40-8*, *GhHsp40-11*, *GhHsp40-20*, *GhHsp40-23*, *GhHsp40-25*, *GhHsp40-53*, GhHsp40-55 were all expressed in *V. dahliae* infection 6 h after dahliae infection, it was significantly higher (Figure 7; Supplementary Figure S5). The expressions of *GhHsp70-2*, GhHsp70-3, *GhHsp70-6, GhHsp70-8*, *GhHsp70-13*, *GhHsp70-19* and *GhHsp70-22* were significantly increased 24 h after inoculation with *V. dahliae* infection (Figure 8; Supplementary Figure S5).

Compared with the control group, *GhHsp40-4*, *GhHsp40-20*, *GhHsp40-23*, *GhHsp40-53*, *GhHsp70-8*, *GhHsp70-13* and *GhHsp70-19* were upregulated under high temperature stress, while GhHsp40-4 was downregulated under high temperature stress (Supplementary Figure S1). Under the condition of low temperature stress, the expression of *GhHsp40-8* and *GhHsp70-19* increased significantly after 1 h and 2 h of treatment (Supplementary Figure S2). Under salt stress, the expressions of *GhHsp40-11* and *GhHsp40-2* were significantly increased after 3 h of treatment, while the expressions of *GhHsp70-19* were significantly increased after 1 h of treatment (Supplementary Figure S3). Under PEG stress, the expressions of *GhHsp40-2* and *GhHsp70-13* increased significantly after 3 h treatment. The expressions of *GhHsp40-11*, *GhHsp70-2* and *GhHsp70-19* were significantly increased after treatment for 1 h. The expression of *GhHsp40-20* was significantly increased after 6 h of treatment (Supplementary Figure S4).

In addition, we also found that the expression of *GhHsp40-2*, *GhHsp40-4*, *GhHsp40-8*, *GhHsp40-11*, *GhHsp40-20*, *GhHsp70-13*, *GhHsp70-19* increased significantly with treatment time under verticillium wilt resistance and various stress treatments. Phylogenetic tree analysis showed (Figure 2) that these Hsp40s were mainly located in Hsp40-I and Hsp40-II subgroups, while *GhHsp70-13* and *GhHsp70-19* were located in Hsp70-III and Hsp70-IV subgroups, respectively. These subgroups may regulate verticillium wilt resistance and abiotic stress response of upland cotton.

## Discussion

Heat shock proteins (e.g., Hsp40 (DnaJ), Hsp60, Hsp70, Hsp90, and Hsp101) are one of the most common chaperones and participate in a variety of biological processes (Guo et al., 2021). In the research on the function of HSPs in animals, researchers found that they can also directly stimulate the cells of the innate immune system, which indicates that they are the activators of the innate immune system of animals (Cui et al., 2011). For example, non-lethal heat shock induces the synthesis of Hsp70, and promotes *Penaeus vanname*i’s tolerance to heat, ammonia and metal induced pressure (Sung et al., 2018). So far, researchers have identified *Hsp40* and *Hsp70* of several plants, such as barley (Landi et al., 2019) and *Brachypodium distachyon (L.) Beauv*. (Wen et al., 2017), *A. thaliana* (Li et al., 2017) and *California poplar* (Yer et al., 2018). Although the functional studies on *Hsp40* and *Hsp70* in plants are limited, these proteins are believed to have multiple roles in plants (Guo et al., 2021) and play similar functions as other eukaryotes.

The Hsp40 protein plays a key role in assisting protein folding by activating the ATPase domain of Hsp70. Despite their importance, the *Hsp40s* and *Hsp70s* of cotton have not been reported. In this study, we identified 291 *Hsp40s* and 171 *Hsp70s* in the genomes of four cotton species. The Hsp40s identified all contain the DNA-J domain to stimulate the ATPase activity of Hsp70, which is essential for the maintenance of Hsp40 function, suggesting that all of these Hsp40s are conserved and can act as copartners of Hsp70s. Sequence analysis showed that there were differences between Hsp40s and Hsp70s in PI and domain combinations. The average PI value of Hsp40s protein is 7.70, while the average PI value of Hsp70 protein is 5.38. One is acidic protein and the other is basic protein, which indicates that acidic protein and basic protein have different positions and functions in cells. The instability index indicates the *in silico* stability of protein (Gonzalez-Diaz et al., 2007), it was found that 41 out of 94 *GhHsp40s* were stable proteins, and the instability coefficient of *GhHsp40-4*, *GhHsp40-38*, *GhHsp40-61* and *GhHsp40-80* were more than 60, indicating that they were easy to degrade. The instability coefficient of most GhHsp70s proteins was less than 40, indicating that GhHsp70s was stable compared with GhHsp40s.

Gene duplication events of entire genomes, chromosomal segments, and individual genes play a role in the expansion of gene family members (Cannon et al., 2004; Maere et al., 2005). These events also contribute to the evolution of novel gene functions and potentially play an important role enhancing plant environmental adaptability (Huang et al., 2015). In order to understand the phylogenetic relationships between *Hsp40s* and *Hsp70s*, and to identify homology relationships within and among four cotton species, we constructed a phylogenetic tree using amino acid sequences of Hsp40s and Hsp70s. According to the phylogenetic tree, the sequence relationship of Hsp40s and Hsp70s can be divided into three groups and four groups respectively. At the same time, we have identified eight pairs of homologous genes in the *Hsp40s* gene family of four cotton varieties, and 14 pairs of homologous genes in the *Hsp70s* gene family. This suggests that the Hsp40s and Hsp70s gene families are species-specific in all four cotton species (Liu et al., 2018).

*Cis*-regulatory elements of promoter region play an important role in the functional differentiation of Hsp40s and Hsp70s genes. Analysis of cis-regulatory elements shows three types of cis-regulatory elements, including hormone responsive elements, stress responsive elements and light responsive elements. Among them, hormone response elements accounted for a large number, such as gibberellin-responsive element, auxin responsive element and abscisic acid responsive element. Plant hormones are important regulators of plant growth and development. Abscisic acid (ABA) regulates the formation of secondary cell wall and lignin deposition in *Arabidopsis thaliana* by phosphorylating NAC Secondary-wall Thicking protein factor 1 (NST1) (Liu et al., 2021). Auxin and gibberellin are promoters of cotton fiber cell elongation (Singh et al., 2009; Xiao et al., 2010). Salicylic acid responsive element and MeJA responsive element, which promote the synthesis and integrity of cell wall through salicylic acid and jasmonic acid metabolism, participate in plant pathogen defense response (Takahashi et al., 2004). Low temperature responsive element, anaerobic induction responsive element and wound-responsive element indicate that *Hsp40s* and *Hsp70s* genes can control environmental conditions. *Hsp40s* and *Hsp70s* may play important roles in different abiotic stress responses due to the existence of multiple *cis*-responsive elements. Therefore, this study provides a theoretical basis for further study of its actual mechanism of action.

There are 7 different domain combinations in the identified Hsp40s. The combination of domains leads to the diversity of cotton Hsp40s functions. The DnaJ domain is a specific feature that defines a protein as Hsp40 or Hsp40-like protein. In addition to the conserved domain, Hsp40 is bound to other domains, which may determine the functional diversity of Hsp40s. For example, tetratricopeptide repeat (TPR) domains, which mediate protein-protein interactions (Dermouche et al., 2021), Studies have shown that the C-terminal of Hsp90 dimer forms a functional receptor site for a co-partner carrying a tetratricopeptide repeat (TPR) domain, and they bind in a mutually exclusive or competitive manner. The biology of the glucocorticoid receptor (Hsp90•GR) complex is more potent, and it is a major player in the GR transport process (Mazaira et al., 2021). Most MYB proteins act as transcription factors and have been shown to be involved in regulating various cellular processes, including cell cycle and morphogenesis, biological and abiotic stress responses (Roy, 2016). In addition to TPR domains, we identified Myb DNA-binding domain in Hsp40 protein, suggesting that Hsp40 protein may bind to MYB transcription factors and be involved in mediating cell development, biological and abiotic stress responses. Compared with Hsp40s, there were only two types of proteins in Hsp70s, indicating that Hsp70 had more conservative functions.

The diversity of domain combinations resulted in the functional diversity of Hsp40s and the conserved function of Hsp70s. By analyzing the transcriptome of *Hsp40s* and *Hsp70s* under low temperature, high temperature, salt and PEG stress, it was found that Hsp40-I, Hsp40-II, Hsp70-III and Hsp70-IV subfamily members may respond to verticillium wilt resistance and abiotic stress. Such as, *GhHsp40-2*, *GhHsp40-4*, *GhHsp40-8*, *GhHsp40-11*, *GhHsp40-20*, *GhHsp70-13* and *GhHsp70-19* showed significantly increased expression levels with the increase of treatment time under verticillium wilt resistance and each stress treatment condition. They play a positive regulatory role in cotton response to verticillium wilt resistance and abiotic stress. This provided a theoretical basis for further study of gene family in resistance to abiotic stress and verticillium wilt resistance.

## Conclusion

In this study, 291 *Hsp40s* and 171 *Hsp70s* gene family members were identified in cotton by bioinformatics. The analysis of their gene distribution and protein structure shows that these two Hsp families have complex evolutionary history. Promoter analysis showed that Hsp40s and Hsp70s were involved in abiotic stress tolerance and verticillium wilt resistance, which was verified by the expression profile analysis of high temperature, low temperature, PEG, salt stress and verticillium wilt resistance. Therefore, this study shows that *GhHsp40-2*, *GhHsp40-4*, *GhHsp40-8*, *GhHsp40-11*, *GhHsp40-20*, *GhHsp70-13*, *GhHsp70-19* are potential candidate genes for further functional analysis in cotton variety improvement research. This study provided a good theoretical basis for further study of the biological functions of *Hsp40s* and *Hsp70s* gene families in cotton.

## Data Availability

The datasets presented in this study can be found in online repositories. The names of the repository/repositories and accession number(s) can be found in the article/Supplementary Material.

## Abbreviation

Hsp, heat shock proteins.
