# Supplementary figures and images for: Genome-wide analysis of Hsp40 and Hsp70 gene family in four cotton species provides insights into their involvement in response to Verticillium dahliae and abiotic stress
Source: Front Genet. 2023 Jan 26;14:1120861. doi: 10.3389/fgene.2023.1120861 (PMC9909605; doi:10.3389/fgene.2023.1120861)

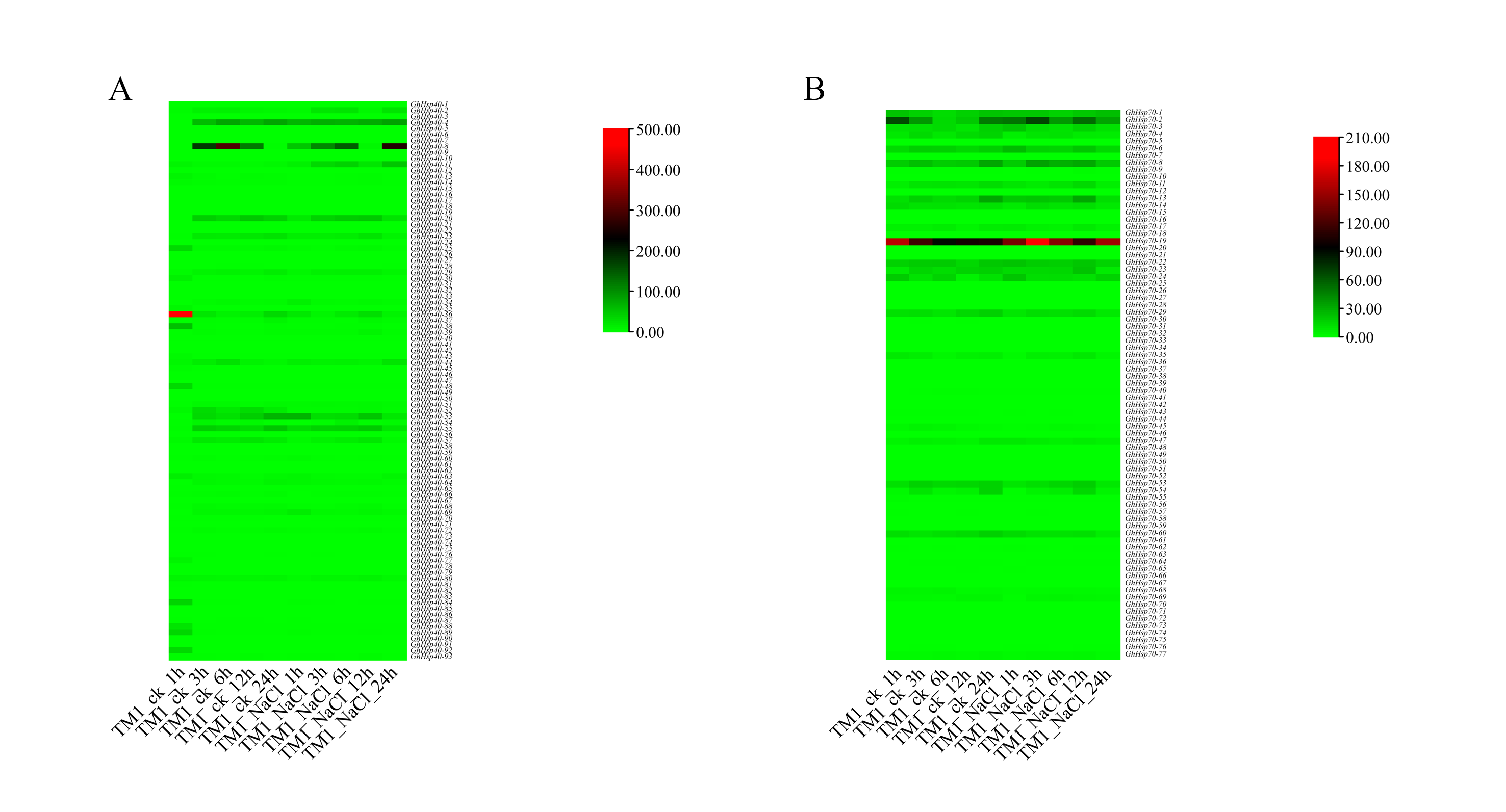

Supplement: Supplementary file 1 [file Image3.tif]

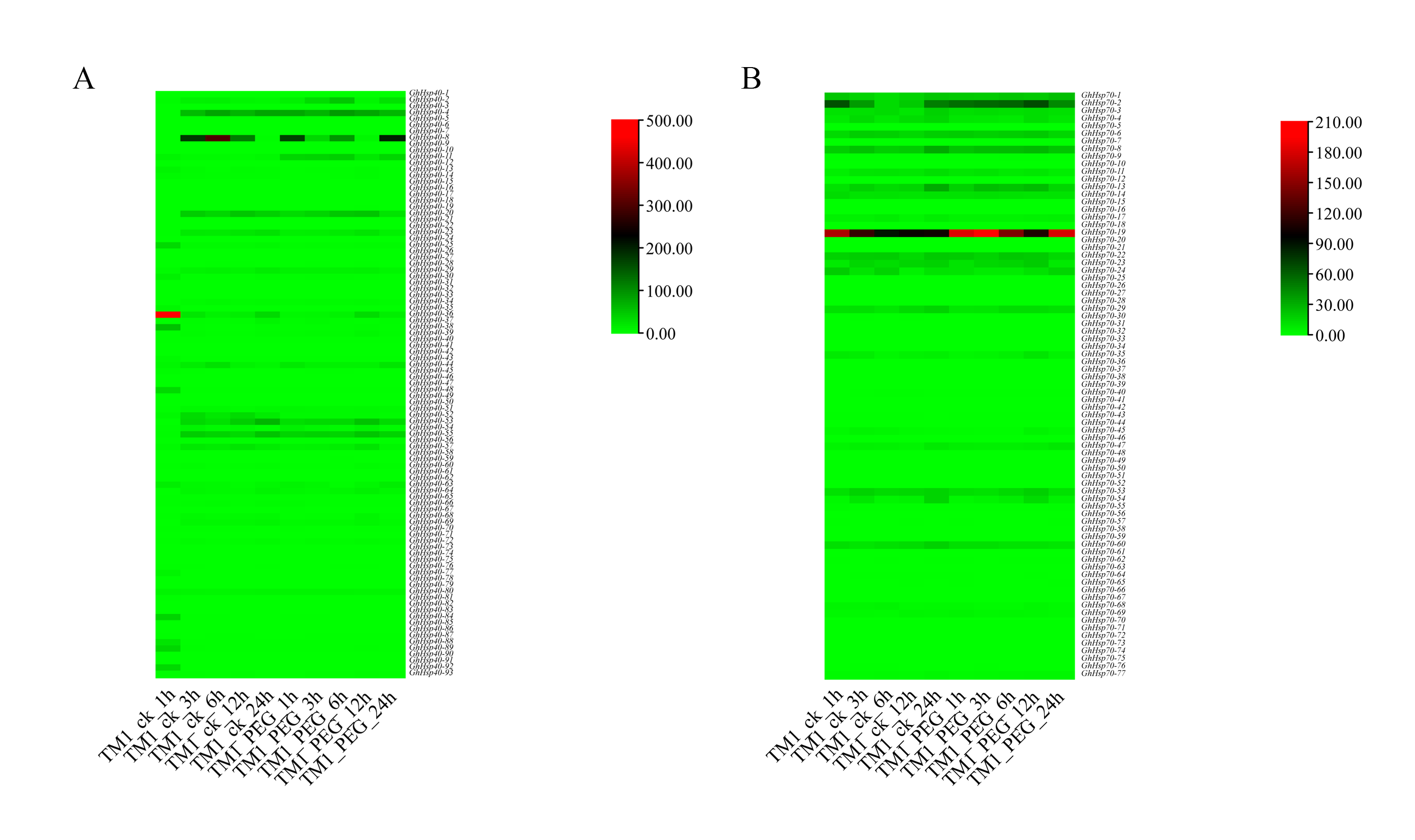

Supplement: Supplementary file 2 [file Image4.tif]

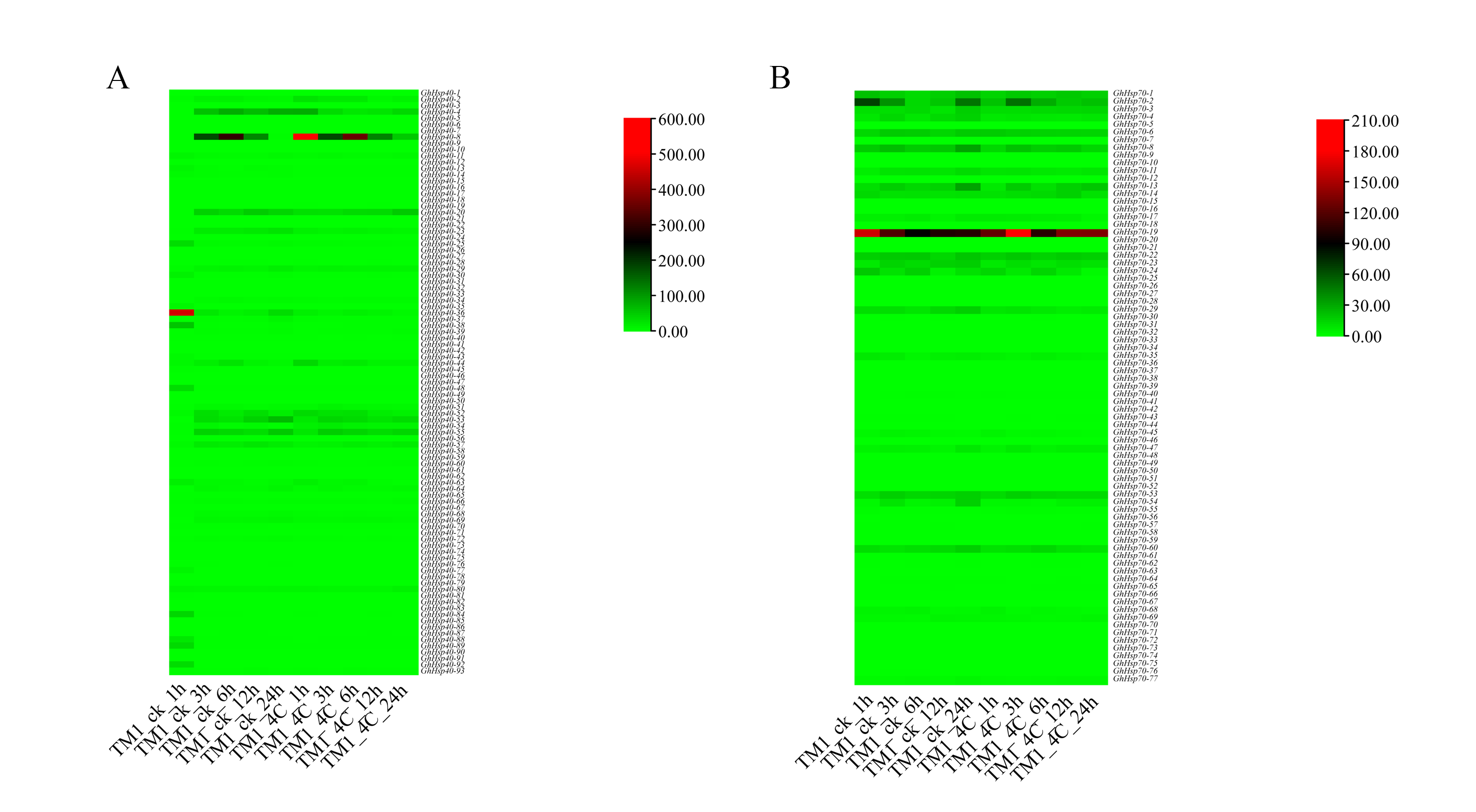

Supplement: Supplementary file 3 [file Image2.tif]

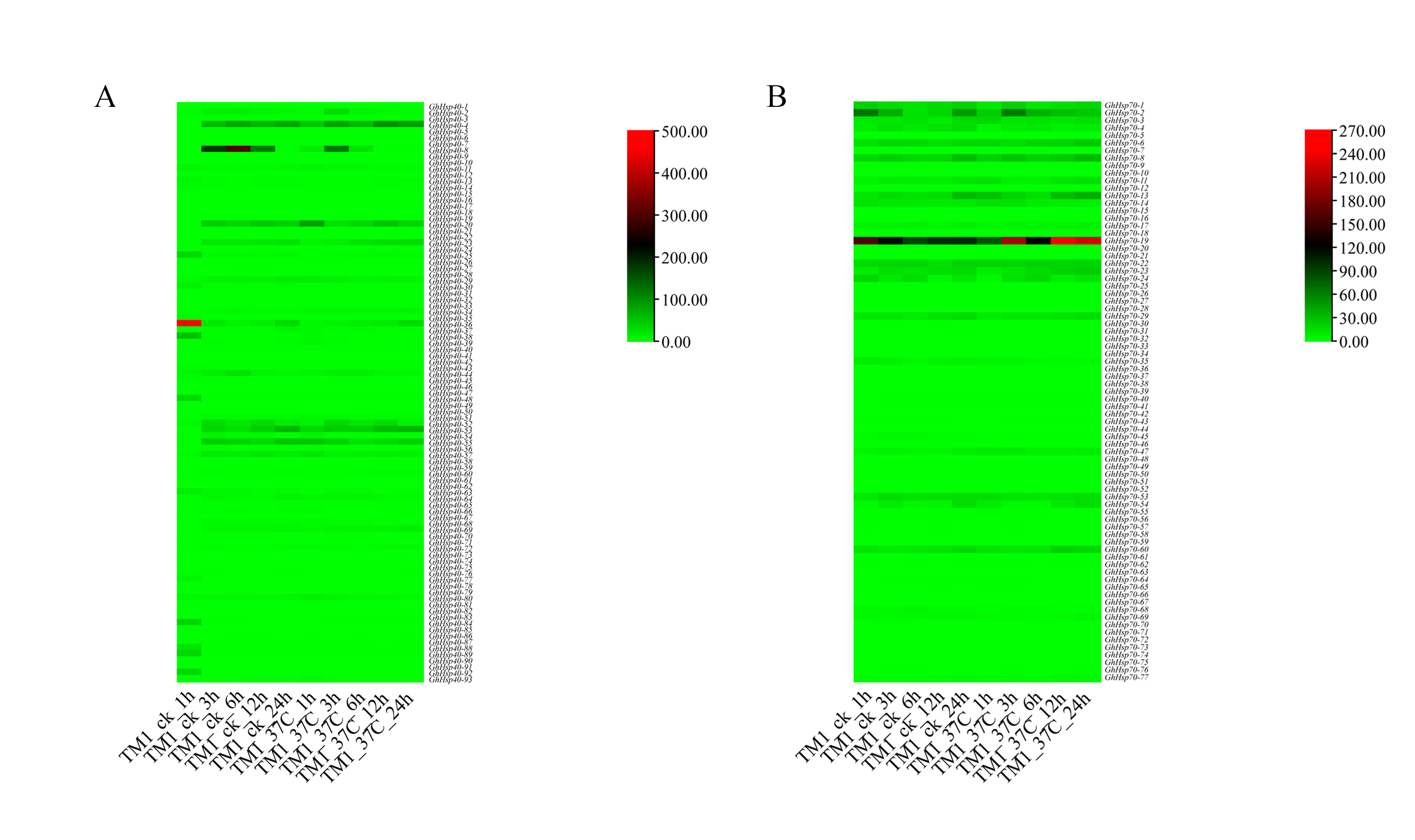

Supplement: Supplementary file 4 [file Image1.tif]

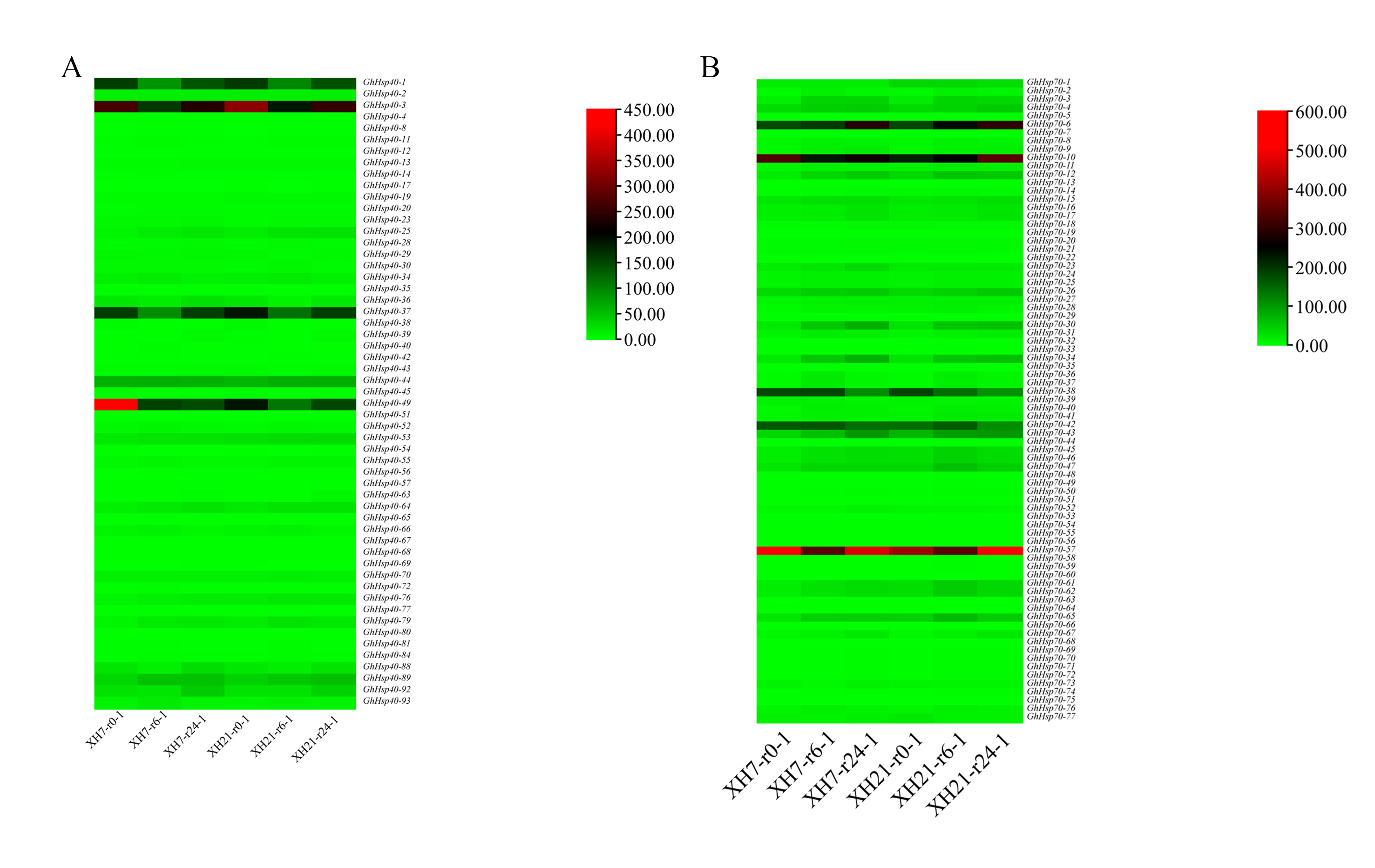

Supplement: Supplementary file 6 [file Image5.tif]
